# Early Developmental and Evolutionary Origins of Gene Body DNA Methylation Patterns in Mammalian Placentas

**DOI:** 10.1371/journal.pgen.1005442

**Published:** 2015-08-04

**Authors:** Diane I. Schroeder, Kartika Jayashankar, Kory C. Douglas, Twanda L. Thirkill, Daniel York, Pete J. Dickinson, Lawrence E. Williams, Paul B. Samollow, Pablo J. Ross, Danika L. Bannasch, Gordon C. Douglas, Janine M. LaSalle

**Affiliations:** 1 Department of Medical Microbiology and Immunology, The University of California Davis School of Medicine, Davis, California, United States of America; 2 University of California Davis Genome Center, University of California Davis, Davis, California, United States of America; 3 University of California Davis MIND Institute, University of California Davis, Sacramento, California, United States of America; 4 Department of Population Health and Reproduction, UC Davis School of Veterinary Medicine, Davis, California, United States of America; 5 Department of Veterinary Integrative Biosciences, Texas A&M University, College Station, Texas, United States of America; 6 Department of Cell Biology and Human Anatomy, University of California Davis School of Medicine, Davis, California, United States of America; 7 Department of Surgical and Radiological Sciences, University of California School of Veterinary Medicine, Davis, California, United States of America; 8 Department of Veterinary Sciences, University of Texas MD Anderson Cancer Center, Bastrop, Texas, United States of America; 9 Department of Animal Science, University of California Davis, Davis, California, United States of America; The Babraham Institute, UNITED KINGDOM

## Abstract

Over the last 20-80 million years the mammalian placenta has taken on a variety of morphologies through both divergent and convergent evolution. Recently we have shown that the human placenta genome has a unique epigenetic pattern of large partially methylated domains (PMDs) and highly methylated domains (HMDs) with gene body DNA methylation positively correlating with level of gene expression. In order to determine the evolutionary conservation of DNA methylation patterns and transcriptional regulatory programs in the placenta, we performed a genome-wide methylome (MethylC-seq) analysis of human, rhesus macaque, squirrel monkey, mouse, dog, horse, and cow placentas as well as opossum extraembryonic membrane. We found that, similar to human placenta, mammalian placentas and opossum extraembryonic membrane have globally lower levels of methylation compared to somatic tissues. Higher relative gene body methylation was the conserved feature across all mammalian placentas, despite differences in PMD/HMDs and absolute methylation levels. Specifically, higher methylation over the bodies of genes involved in mitosis, vesicle-mediated transport, protein phosphorylation, and chromatin modification was observed compared with the rest of the genome. As in human placenta, higher methylation is associated with higher gene expression and is predictive of genic location across species. Analysis of DNA methylation in oocytes and preimplantation embryos shows a conserved pattern of gene body methylation similar to the placenta. Intriguingly, mouse and cow oocytes and mouse early embryos have PMD/HMDs but their placentas do not, suggesting that PMD/HMDs are a feature of early preimplantation methylation patterns that become lost during placental development in some species and following implantation of the embryo.

## Author Summary

The placenta is vital for the proper development of the fetus, not only facilitating the exchange of nutrients, oxygen, and waste between the mother and the fetus but also acting as an interface to the maternal immune system and regulating fetal growth by excreting hormones and growth factors. DNA methylation is important for both placental and embryonic development as loss of proteins involved in DNA methylation can result in placental dysmorphology and early embryonic death. The human placenta has a unique DNA methylation landscape characterized by alternating regions of low methylation, covering silent genes with tissue-specific developmental functions, and high methylation, covering active genes. In order to better understand the significance of this DNA methylation landscape in the human placenta, we performed a cross-species comparison of DNA methylation in mammalian placentas, oocytes, and early embryos from this and other studies. Although the levels and extent of hypomethylation differed between mammalian placentas, what we found to be highly conserved was relatively higher methylation levels over active genes. These same genes also had high methylation in the opossum extraembryonic membrane, a primitive placenta, as well as oocytes and early embryos, suggesting that high methylation over these genes predated placental mammals and is established very early in development.

## Introduction

In eutherian mammals the placenta plays a vital role in not only the transfer of nutrients and waste between mother and offspring but also as a protective layer between the maternal and fetal immune systems during fetal development. Despite this, the gross morphologies and cellular characteristics of the maternal/fetal interface are quite diverse [1,2] and have undergone multiple instances of both divergent and convergent evolution [3]. Marsupials also have an extraembryonic membrane (EEM) that, although short-lived, is also important for nutrient exchange, originates from a trophectoderm layer in the early embryo, and has been argued to be a true placenta [4,5,6,7].

DNA methylation is essential for proper embryo and placenta development. The offspring of *Dnmt3a* conditional knockout mothers die *in utero* by E11.5 [8]. The offspring of *Dnmt3L* null mothers die at E9.5 from placental abnormalities and/or imprinting defects [9,10,11]. Another DNMT important for normal placenta development is DNMT1o, an oocyte-specific isoform of DNMT1 that is present in eutherians as well as metatherians [12]. Loss of *Dnmt1o* in mice results in widespread placental dysmorphology [13,14].

It has long been known that human and mouse placentas are hypomethylated compared to other tissues [15,16,17,18,19]. However, recent analysis of human placenta has shown a large-scale pattern of PMDs and HMDs that are often over 200 kb in length and can cover entire genes and gene clusters [20,21]. Human placenta PMDs cover tissue-specific genes that are transcriptionally repressed. These findings provide a unique opportunity to use DNA methylation to study not only the evolution of transcriptional regulation in this developmentally important organ but also the molecular similarities between species with morphologically distinct placentas. One study has shown that gene-specific methylation in mammalian placentas tracks with phylogeny more than placental morphology [22], but otherwise little is known about the evolution of DNA methylation and transcriptional regulation in placenta.

The placenta derives from the trophectoderm layer of the blastocyst in the early embryo, before the implantation stage. The DNA methylation patterns of the early embryo have recently been elucidated. Human and mouse oocytes have low levels of methylation which decrease even further in the fertilized embryo until after the blastocyst stage [23,24,25,26,27]. Therefore, it is thought that the placenta may never undergo the wave of remethylation that occurs in cells with other somatic tissue fates [28].

We performed MethylC-seq on a representative set of mammalian placentas, both with respect to evolutionary relationship and placental morphology. We found that although PMD/HMDs were not found in the placentas of many species, what was conserved was high methylation over gene bodies, particularly over those of active genes. Thus, in many mammalian placentas high methylation is found over single genes instead of over entire clusters of genes, as is the case in placentas with PMD/HMDs. Low genome-wide methylation and high gene body methylation in transcriptionally active genes was also found in the opossum EEM, suggesting that this is a conserved feature in the evolution of the mammalian placenta. Finally, analysis of DNA methylation in oocytes and preimplantation embryos shows that low global methylation but higher methylation over gene bodies is present prior to fertilization and persists through the blastocyst stage

## Results

### Mammalian placentas differ in their levels and patterns of global DNA methylation

**Fig 1:**
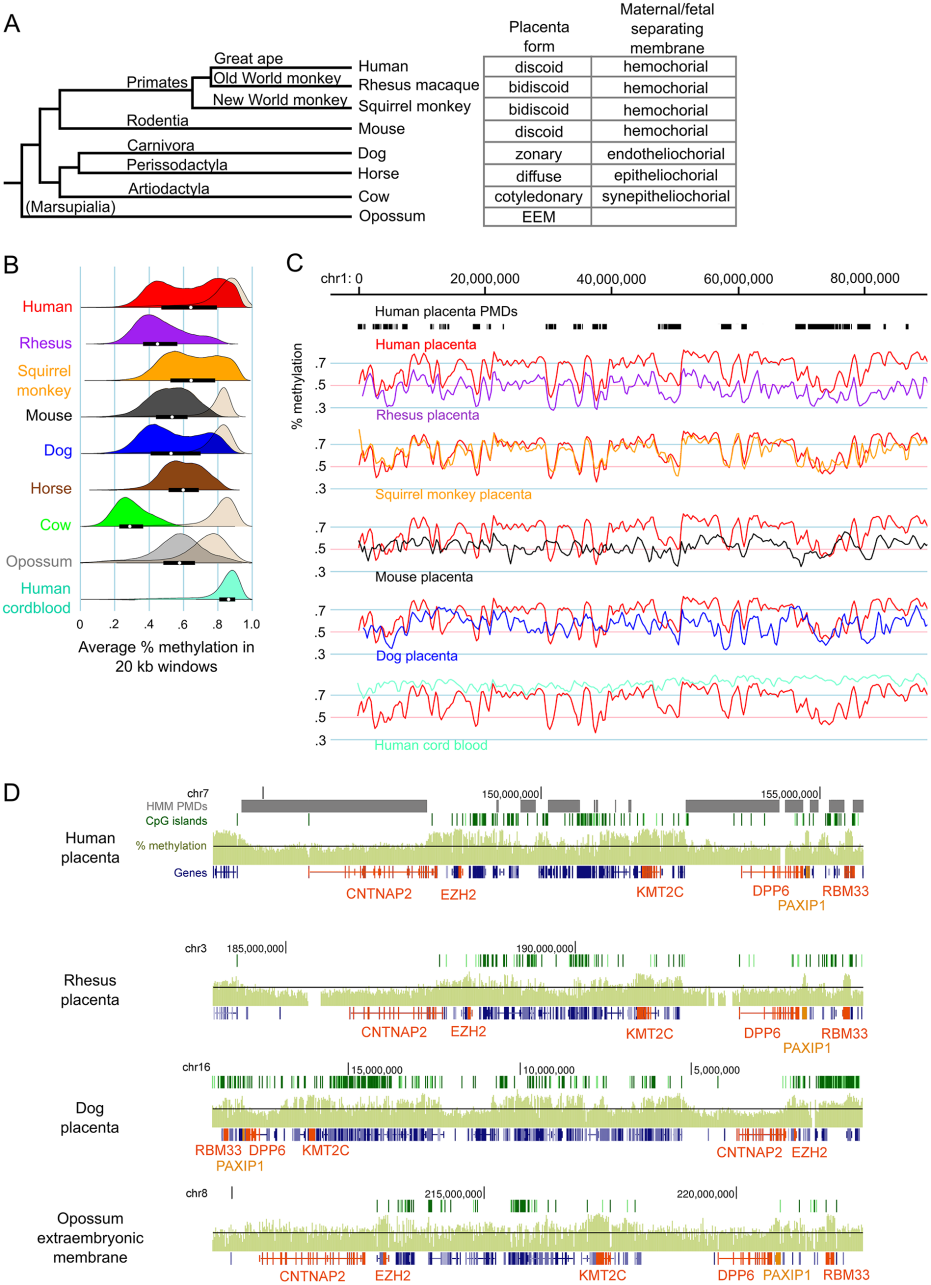
Genome-wide methylation patterns in mammalian placentas show both large-scale divergence and gene-specific similarities. (A) Phylogenetic tree of the species studied and the classification of their placenta types. Branch lengths are not to scale. (B) Density curves of average percent methylation in non-overlapping 20 kb windows in mammalian placentas. For comparison, the methylation distribution for brain tissue is shown in beige for some species and human cord blood is shown in aquamarine. The opossum brain sample was fetal whereas the other brain samples were postnatal. The interquartile range and medians are shown as black bars and white dots, respectively, for the placenta samples. Mouse brain data is from Hon et al. [19] (GSE42836), human placenta data is from Schroeder et al [21] (GSE25930). (C) Comparison of global methylation patterns in select species after liftOver to the human genome and smoothing (for full figure, see S4 Fig). Human placenta PMDs, as determined by HMM, are shown in black bars at top. (D) Comparison of methylation patterns at the *CNTNAP2* locus in select species (for full figure, see S5 Fig). Raw species CpG site methylation wig data were graphed on the UCSC Genome Browser without preprocessing. Black line represents 50% methylation. Genes of interest are orange. EEM = extra-embryonic membrane.

Low coverage MethylC-seq was performed on rhesus, squirrel monkey, mouse, dog, cow, and horse placentas as well as opossum EEM (S1 Table). Fig 1A shows the phylogenetic relationship between the species in this study and the type of placental morphology in each.

In order to compare the pattern of DNA hypomethylation across mammalian placentas, average methylation was calculated for non-overlapping 20 kb windows tiled across the autosomes for each species. To compare placenta methylation levels to those of a somatic tissue, we also performed MethylC-seq on adult cow and dog cerebrum samples as well as fetal opossum whole brain. Fig 1B shows that mammalian placentas vary greatly in their overall amount of DNA methylation, although all were globally hypomethylated compared to brain, including the opossum EEM. All placentas had lower than 66% average methylation (calculated as the average methylation of all CpG sites in all reads that aligned to the genome), but cow in particular had a remarkably low level of methylation in placenta (30%), even though cow brain global methylation is comparable to that of other mammals. In spite of this, methylation levels in regulatory elements such as CpG islands and promoters are similar between species (S1 Fig) and repetitive elements in general follow similar patterns, with LINEs and LTRs having methylation levels similar to those of non-repetitive regions and SINEs having relatively higher methylation (S2 Fig).

While human, squirrel monkey, and dog placentas show a bimodal distribution of methylation levels indicative of PMD and HMDs, other species showed a single peak or, like rhesus, had evidence of a small number of HMDs seen as a small secondary peak at higher methylation values. These results were similar for window lengths of 5–50 kb (S3 Fig). Since the species showing PMD/HMD divisions were not necessarily the most closely related to humans, we next examined the conservation of methylation patterns at the chromosome and gene level. Homologous chromosomal regions of each species were mapped to the human genome using the liftOver program (see Methods). Fig 1C shows that, despite the weak evidence for PMD/HMDs in the rhesus methylation density curves, methylation patterns across the rhesus chromosomes follow the patterns in human placenta remarkably well, with a Pearson correlation of 0.69 in 20 kb windows, the highest of all the species in this study (S2 Table). Interestingly, despite the vast differences in the medians and interquartile ranges of window methylation levels between the different species, the correlations are remarkably high, with mouse placenta having the least similarity with human placenta of the species studied (S2 Table and Figs 1C and S4).

Mapping a species genome to the human genome can introduce errors and artifacts, particularly in distantly-related species such as opossum (S4 Fig). To eliminate these we next examined two loci with high methylation conservation between the species without mapping to the human genome (Figs 1D, S5 and S6). Figs 1D and S5 show the *CNTNAP2* locus which includes two genes involved in neuronal development, *CNTNAP2* and *DPP6*, as well as the gene encoding Polycomb group protein EZH2. Since neuron-specific genes tend to be in PMDs in human placenta [21], we asked if hypomethylation was a conserved feature of this neuronal gene locus in the placenta. Figs 1D and S5 show that in most species both *CNTNAP2* and *DPP6* are in regions of relatively lower methylation compared to the surrounding locus. Most striking, however, are the short regions of relatively high methylation over individual genes or small clusters of genes that are highly conserved across all species, including the opossum EEM. *EZH2*, *KMT2C* (a histone methyltransferase expressed in placenta), *PAXIP1* (a gene that maintains genome stability during mitosis), and *RBM33* (a hypothetical RNA-binding protein) have higher than average methylation in all the species studied. Likewise in the *DAB1* locus (S6 Fig), genes such as *USP24* (a ubiquitin peptidase), *FGGY* (phosphorylates carbohydrates), *INADL* (scaffolding protein in the cell membrane), *DOCK7* (a guanine nucleotide exchange factor), and *ATG4A* (cysteine protease required for autophagy) are highly methylated in most species.

Our previous MethylC-seq analysis of three full-term human placentas showed that PMDs and HMDs were highly reproducible across individuals and gestational ages [21]. Here we performed five additional experiments to test the sensitivity of placenta methylome data to sampling differences and cellular heterogeneity. First, MethylC-seq analysis of whole rhesus placental tissue and isolated rhesus trophoblast cells gave nearly identical results with a correlation of 0.89 (S7A, S7B, and S7C Fig). MethylC-seq results for E15.5 C57Bl6/J mouse placenta are very similar to those previously reported by Hon et al. [19] and a E11.5 C57BI6/J placenta sample (S7D, S7E and S7F Fig) with pairwise correlations over 0.87. Since cow placenta has such low methylation, to validate the results we sequenced three additional biological replicates using tissue material from a different source and found nearly identical results with correlations above 0.75 (S8A, S8B, and S8C Fig). We also sequenced two additional dog placentas from different breeds and developmental timepoints (S1 Table). Methylation patterns across the chromosomes were very similar and pairwise correlations were over 0.87 (S8D and S8F Fig). Although one of the dog placentas did not show a bimodal distribution whereas the other two did (S8E Fig), the relative pattern of methylation along chromosomes was highly similar and the pairwise correlations were high (0.89 and 0.87). Thus, the length of the range of methylation values in the curve may be more important than its bimodality. Finally, to confirm the reproducibility of human placenta MethylC-seq across samples, labs, and sequencing coverage, we compared our three previously sequenced placentas [21] to that of a higher-coverage MethylC-seq sample [29]. S9 Fig shows that all four human placenta samples have nearly identical global methylation patterns with pairwise correlations of >0.95, demonstrating that sequencing depth does not affect global methylation levels or patterns.

### High methylation is associated with the gene bodies of actively transcribed genes

**Fig 2:**
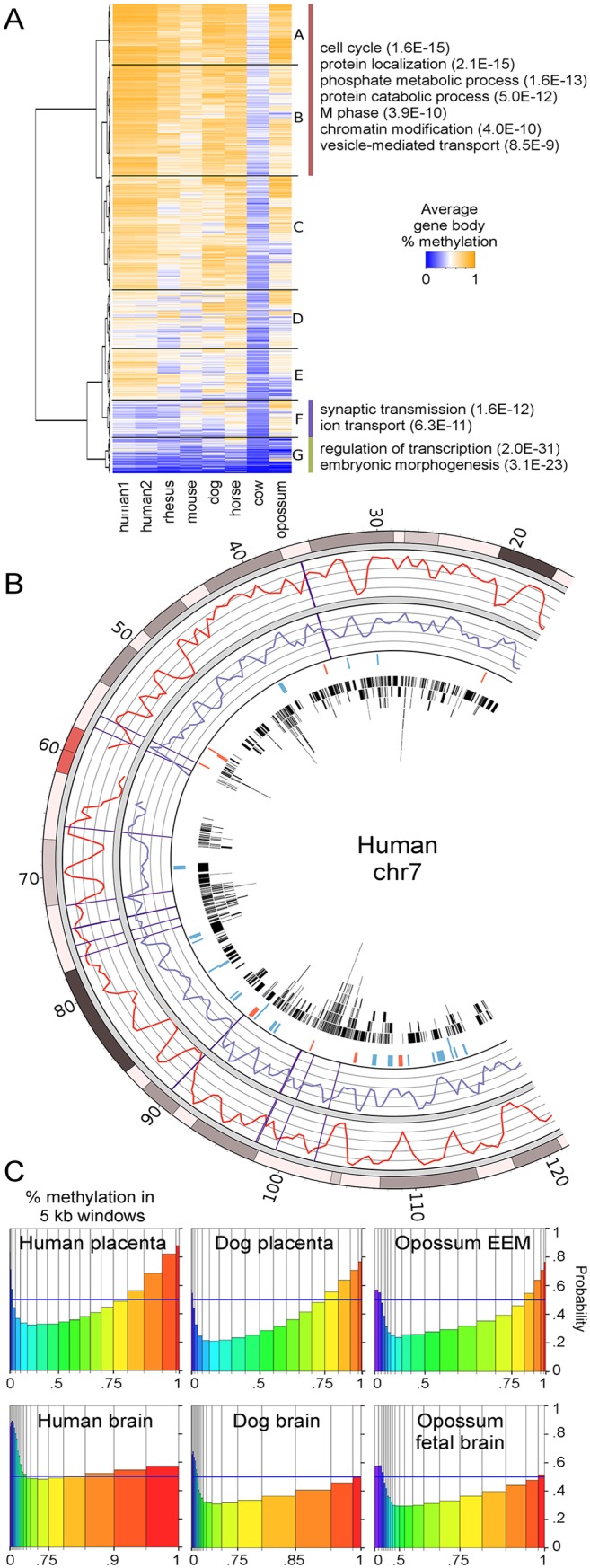
Regions of high methylation in placentas cover gene bodies. (A) Heatmap of average methylation in the gene bodies (introns and exons, excluding CpG islands and promoters) of orthologous genes. Only the top few GO biological processes with Benjamini p-values below 1.0E-3 are shown. For a complete list see S3 Table. Branches A and B were combined because they contain similar GO terms. (B) Comparison of percent methylation between human placenta (red curve) and rhesus placenta (purple curve). Rhesus methylation data was lifted over to the human genome. Vertical purple lines show large chromosomal breaks in synteny between the two species. The fourth ring in shows regions of higher (blue) and lower (red) methylation in human placenta compared to rhesus. The fourth circle in shows the locations of human genes in black. (C) Spinograms showing the probability that a 5 kb window is in a gene given that window's average percent methylation. Bars are color-coded by percent methylation and bar widths show the percentage of windows with that methylation level. Bars furthest from the 0.5 blue line marker show the most information about gene location.

To determine the functions of genes with conserved high methylation in mammalian placentas, orthologous Ensembl genes in the seven species were clustered based on average gene body methylation (Fig 2A). We found a group of 3380 genes (branches A and B) that had relatively high methylation within each species. This group is enriched for functions such as cell cycle, protein localization, and protein ubiquitination (S3 Table). In contrast, genes with consistently low methylation across species tended to be transcription factors and have developmental functions (branch G), of which 30% are polycomb-regulated. Our previous study found that human placenta also has low methylation over some tissue-specific genes such as those involved in neuronal functions of synaptic transmission and ion transport [21]. Across species, however, this trend of lower methylation of neuronal genes is not as conserved since dog, horse, and opossum show mixed or higher levels of methylation in these genes (branch F).

An interesting question is whether conservation of long-range methylation patterns (eg. human and rhesus in Fig 1C) is due to conservation of large chromatin domains or whether such patterns arise at the gene level. To address this question, we compared regions with large chromosomal breaks in synteny with regions of differential relative methylation between human and other species (S10A Fig). Fig 2B shows a portion of human chromosome 7 with a relatively large number of chromosomal syntenic breaks between human and rhesus. Syntenic breaks are not enriched for differential methylation, suggesting that methylation patterns are established at a more local level.

Since we had shown that DNA methylation was most conserved in gene bodies, we examined more closely the relationship between DNA methylation and genes. S10B Fig shows that the boundaries of human placenta PMDs are closer to gene ends and CpG islands than expected by chance. We next asked if DNA methylation is predictive of gene location in the placentas of all species studied. Non-overlapping 5 kb windows were tiled across the autosomes of each species and classified as genic or intergenic. S11 Fig shows that intergenic regions have lower DNA methylation levels than genic regions. Figs 2C and S12 show the probability that a window is in a gene based on its average % methylation. The further the probability is from 0.5, the more informative the methylation level is in predicting the presence (above) or absence (below) of a gene. In all species studied, including opossum, high placental methylation was associated with genes, as was very low methylation since CpG islands were not removed. This is in contrast to brain where high methylation gives little to no information about the presence of a gene. Together these data indicate that DNA methylation patterns in placenta are being set at the gene level.

**Fig 3:**
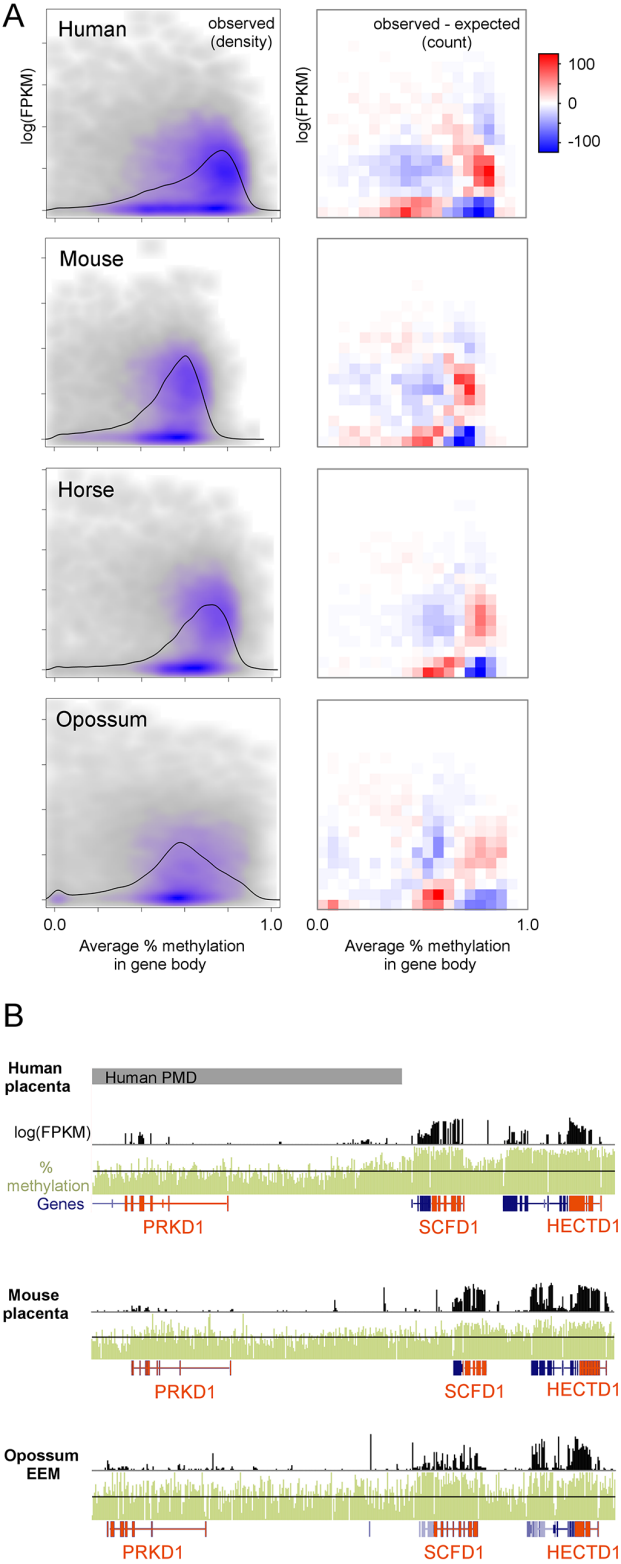
Regions of high methylation are enriched for actively transcribed genes. (A) Relationship between average gene body percent methylation and gene expression in placentas. Only gene with orthologs were used. The right column shows a density scatterplot with gray and purple show areas of low and high density, respectively. Black lines show the marginal distribution of percent methylation in gene bodies. The left column shows the number of genes in each quadrant above/below what would be expected if gene expression and gene body methylation were independent. Human, mouse, and opossum expression data are from Necsulea et al. [30] (GSE43520) and horse expression data is from Wang et al. [31](GSE30243). (B) Comparison of methylation and gene expression patterns at the *HECTD1* locus. Genes of interest are orange.

We next asked whether, as in human placenta, genes with high methylation are more likely to be expressed across a broad range of mammals with diverse placental anatomies [21]. Published polyA-selected RNA-seq data for human, mouse, and horse placenta and opossum EEM [30,31] were utilized to compare gene expression to gene body methylation in orthologous genes. In human placenta, expressed genes have clearly higher than average gene body methylation levels (Fig 3A, left column). To a lesser extent this can also be observed in mouse, horse, and opossum, although the range of gene body methylation values is smaller than human. To determine if these distributions are different than we would expect by chance given the marginal gene body methylation and gene expression distributions, we divided the x and y axes into 20 equally-spaced bins, counted the number of observations in each resulting quadrant, and compared that to the expected number of observations if gene body methylation and gene expression were independent (Fig 3A, right column). A co-independence test showed a statistically significant deviation from independence for all four species, but more importantly the patterns of deviation are remarkably similar between the species. In all species examined, genes with high gene body methylation are more likely to have intermediate expression than expected by chance and genes with low methylation are less likely to be expressed.

An example of the overlap between methylation and expression is the *HECTD1* locus in Fig 3B. The *HECTD1* gene encodes an E2 ubiquitin protein ligase that is important for proper placenta development in mouse [32]. *HECTD1* has higher than average methylation levels and is expressed in human, mouse, and opossum. This is also true for the neighboring gene *SCFD1*, which encodes a protein involved in SNARE-pin assembly and vesicular transport.

### Cow and mouse oocytes show gene body methylation patterns similar to those found in mammalian placentas

**Fig 4:**
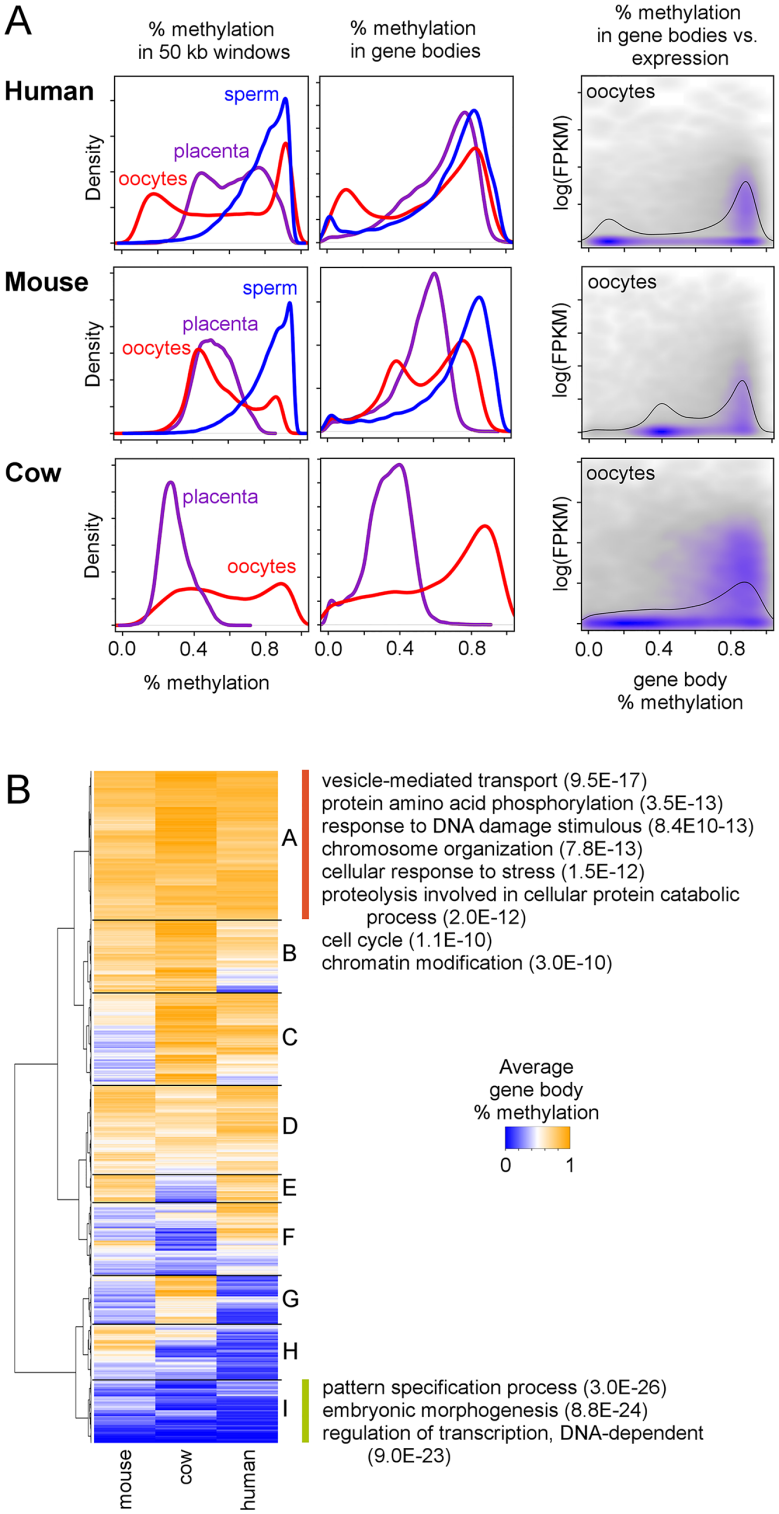
Gene methylation and expression in oocytes compared to placenta in mouse and cow. (A) Distribution of average methylation in 50 kb windows (first column) and gene bodies (second column) during early human, mouse, and cow development. The third column shows the relationship between average gene body methylation and gene expression in oocytes. Black lines show the marginal distribution of percent methylation in gene bodies. All protein-coding genes from each species were used. Human oocyte and sperm methylation data are from Okae et al. [27] (JGAS00000000006), human oocyte expression data are from Reich et al. [34] (GSE32689), mouse oocyte and sperm methylation and expression data are from Wang et al [24] (GSE56697), and cow oocyte expression data are from Graf et al [33] (GSE52415). (B) Heatmap of average gene body methylation in human, mouse, and cow oocytes. For a complete list of all GO and KEGG terms for each quadrant, see S3 Table.

An important question is whether mammalian placentas inherit their distinct methylation patterns from the early embryo or the patterns instead emerge later during placental development. To answer this question we utilized a combination of mouse oocyte and early embryo methylation data [24] and human oocyte data [27] and also performed MethylC-seq on MII cow oocytes. Similar to what was observed in mouse and human oocytes [23,27], cow oocytes exhibit a bimodal methylation distribution (Figs 4A, left column, and S13), a surprising result since mouse and cow placentas do not show bimodal distributions. In addition, although both human oocytes and placenta have bimodal methylation distributions, the global patterns are quite dissimilar (S14A Fig).

We next looked at gene body methylation. A bimodal distribution of average gene body methylation was also observed in all three species' oocytes, although this is more prominent in the human and mouse oocytes (Fig 4A, center column). In mouse, where MethylC-seq data is available for multiple early developmental timepoints, gene body methylation shows bimodal distributions that persist through the inner cell mass (ICM) stage (S13 Fig) and gene body methylation patterns remain correlated between the oocyte and ICM stages (S15 Fig) suggesting that although global methylation levels drop, relative levels of methylation in gene bodies remains consistent.

Using oocyte expression data [24,33,34] we found that, similar to what was seen in placenta, methylation is enriched in gene bodies in oocytes and the mouse early embryo (S16 Fig) and genes with high gene body methylation are more likely to be expressed (Fig 4A, right column). Genes with high methylation in the oocytes of all three species are enriched for functions related to vesicle-mediated transport, protein phosphorylation, and chromosome organization (Fig 4B and S3 Table), similar to that seen in mammalian placentas (Fig 2A and S3 Table). In fact, genes with high expression in both placenta and oocytes are enriched for these same functions (S17A and S17B Fig) and there is a large amount of overlap between the genes that are highly methylated in placenta and oocytes and those that are highly expressed in placenta and oocytes. Thus, these genes appear to maintain a similar regulatory pattern in the placenta that was set up very early in development.

## Discussion

In this first comparative study of genome-wide methylation patterns in mammalian placentas, we have made several novel findings of relevance to understanding the association of transcription and methylation in early life. First, we confirm that hypomethylation of extraembryonic tissues compared to somatic tissues is observed across eutherian mammals and a metatherian mammal as well. Second, we demonstrate that while large differences exist between mammalian placentas in global methylation levels, higher relative methylation of active genes is an evolutionary conserved feature. Third, we show that relative methylation is predictive of genic location in placenta. Lastly, we establish that the pattern of higher gene body methylation of active genes is also observed in mammalian oocytes and persists in the preimplantation embryo.

While hypomethylation of placenta and EEM compared to somatic tissue was observed across mammalian species, global levels of placental methylation and the presence/absence of a bimodal PMD/HMD organization was diverse. The diversity of global methylation levels in mammalian placentas is not entirely surprising given the diversity of mammalian placenta morphologies. Based on current models of the mammalian radiation, it would appear that PMD/HMDs arose or were lost multiple times during placental evolution. However, our analysis of mouse and cow oocytes shows that even though some species do not have PMD/HMDs in their placentas, PMD/HMDs may still exist in the oocytes and early embryos, suggesting that these methylation patterns are later lost during placenta development in those species.

One potential limitation of this study is the inherent diversity of placental morphologies across species and issues of cellular heterogeneity. Differences in absolute methylation levels and the existence of PMD/HMDs could have to do with these confounding factors rather than real differences between species. However, analysis of placental biological replicates in multiple species shows that while absolute global levels of methylation may vary up to 20% due to tissue sampling and inter-individual differences, the methylation patterns across a chromosome remain remarkably similar. Thus, our conclusion of relatively higher methylation over active genes as a conserved feature of mammalian placentas is unaffected. Another potential limitation is the relatively low MethylC-seq coverage used in this study, as low as 1X coverage for most of the placentas used in this study (S1 Table). However, Ziller et al. experimentally determined coverage recommendations for whole-genome bisulfite sequencing, demonstrating that 1X coverage was sufficient for analysis of differentially methylated regions over 5 kb in length with methylation differences over 20% given two sequencing replicates [35].

One interesting question is why PMDs are hypomethylated. It was previously found in IMR90 fetal lung fibroblast cells that PMDs have a unique methylation distribution at individual CpG sites compared to HMDs [36]. High-coverage MethylC-seq data showed that within PMDs, each CpG site has a seemingly random level of methylation, but with some correlation in methylation between neighboring CpG sites less than 100 bp away. Moreover, the best predictor of the methylation level at a CpG site in PMDs was the distance to the nearest neighboring CpG site. In PMDs, unlike in CpG islands, CpG density is positively correlated with methylation. The authors hypothesize that PMDs may represent regions with reduced access to DNMTs and that in such an environment subtle DNMT sequence binding preferences may be easier to detect.

Why do extraembryonic and placental tissues exhibit low global methylation and high gene body methylation, but somatic tissues such as brain are globally highly methylated? One hypothesis stems from the observation that hypomethylation and well-defined PMD/HMD structures are also found in cancerous tumors [37,38,39]. Remarkably, the presence of hypomethylated PMDs is the most epigenetically defining feature of a wide range of human solid tumors [40], EBV transformation of B cells is characterized by the gain of hypomethylated PMDs [41], and brain tumors gain hypomethylated PMDs as they progress to higher metastases [42]. Since placenta shares many features in common with metastatic tumors, including rapid proliferation, invasiveness, and angiogenesis [43,44,45], perhaps a hypomethylated state would be evolutionary advantageous in a temporary tissue in which growth is highly regulated by pregnancy hormones. The temporary nature of the placenta may also make is less susceptible to the effects of retrotransposable elements. In contrast, somatic tissues in more long-lived mammalian species may have evolved globally higher methylation levels as a mechanism to prevent cancer in other tissues.

## Methods

### Tissue sources

The sources of the placenta and brain samples sequenced for this study are listed in S1 Table. Human subjects were approved by the UC Davis IRB (225645–17) and maternal written consent was obtained. All procedures involving animals were performed in accordance with the NIH Guide for the Care and Use of Laboratory Animals and under the approval of the University of California Davis, Animal Care and Use Committee (Animal Protocol #15639). Where possible, villus tissue was taken from the fetal side or interior (equidistant from the fetal and maternal sides) of the placenta, excluding large blood vessels, membranes, and any obvious connective tissue. Opossum EEM tissue was collected as described [46]. Samples were stored frozen at -80 degrees Celsius. Based on similarly processed and stored samples of human fetal side placenta, maternal cell contamination is expected to contribute <10% to methylation and sample location and inter-individual differences are expected to contribute to up to 20% differences in percent methylation over PMDs [21].

### Rhesus trophoblast cells

Trophoblast cells were isolated from Rhesus monkey (Macaca mulatta) placental tissue (Gestation Day: 40–65 days) using procedures we have previously described [47,48,49]. All procedures involving animals were performed in accordance with the NIH Guide for the Care and Use of Laboratory Animals and under the approval of the University of California Davis, Animal Care and Use Committee (Animal Protocol #15639). These cells are 95% cytokeratin 7-positive and 5% vimentin-positive, consistent with a predominantly trophoblast population.

### MII stage cow oocytes

MII oocytes were produced by in vitro maturation of GV oocytes collected from slaughter house derived ovaries according to standard protocols. Oocyte maturation was confirmed by presence of a polar body. Then, zona pellucida and first polar body were removed by incubation in 0.5% pronase solution for 2 minutes and vigorous pipetting. Zona-free oocytes were snap frozen in liquid nitrogen and stored at -80°C until DNA extraction.

### MethylC-seq

DNA from rhesus, cow, horse, dog, squirrel monkey, and mouse placenta tissue was purified using Qiagen's Puregene kit. MethylC-seq libraries were made as described previously [21]. Briefly, the genomic DNA was sonicated to ~300 bp and methylated Illumina adapters were ligated to the ends. The library was bisulfite converted, amplified for 14 cycles, and sequenced on either an Illumina HiSeq or GAII. For the opossum EEM, rhesus trophoblast, human cord blood, and cow, dog, and opossum brain samples, MethylC-seq libraries were made using the Epicentre EpiGnome Methyl-Seq kit according to the manufacturer's recommendations except that 14 cycles of amplification were performed. For cow oocytes, DNA from 100 cells was purified using Zymo's Quick-gDNA MicroPrep kit and the MethylC-seq libraries were prepared using Zymo's Pico Methyl-seq Library Prep Kit according to the manufacturer's instructions. After sequencing on HiSeq2500/2000 machines, reads were mapped to the respective genomes using BS Seeker [50] and only one read per genomic position was kept to prevent clonal PCR amplification biases. CpG site methylation data were combined from both DNA strands. Because methylation data was analyzed over large genomic distances and/or smoothed, no minimum coverage was required for CpG sites used in this analysis [35].

### Mapping MethylC-seq data to the human genome and determining chromosomal synteny

Individual CpGs were mapped to coordinates on the human genome using the liftOver program and species-specific liftOver chain files available on the UCSC Genome Browser [51]. Since our analysis focused on global methylation patterns, not individual CpG sites, mappings were done regardless of whether the CpG site was conserved in human. Since our analysis was focused on methylation in large syntenic regions, we removed CpG data in small inter- and intrachromosomal translocations, duplications, etc. using the species-specific synteny net files available on the UCSC Genome Browser. CpGs were removed if they were in the second level fill of the synteny net files and were less than 1 Mb in length. For graphing the cross-species global methylation patterns, these “cleaned” species liftOver data and raw human data were compressed into averages of non-overlapping 20 kb windows (windows with less than 20 CpG sites with methylation information were discarded) and smoothed in R using a kernel smoother.

For graphing the cross-species syntenic breaks, only the breaks between the large syntenic regions in the first level of the UCSC synteny net files and those between large (>1 Mb) syntenic regions in the second level were used since smaller non-syntenic regions had been removed from the methylation datasets. Due to the fact that the squirrel monkey genome (saiBol1) was not yet assembled into chromosomes, many of the “syntenic breaks” between human and squirrel monkey are actually between contigs that may or may not be on the same squirrel monkey chromosome.

### Defining regions of differential methylation across species

After mapping species methylation data to the human genome, the data was smoothed and normalized. Average methylation was taken for non-overlapping 20 kb windows and a running median was computed using a width of 15 windows. The data was then scaled so that each species had the same mean and standard deviation. The smoothed and scaled methylation values were subtracted from the smoothed and scaled human methylation values and regions of differential methylation were defined as those with methylation differences over 1.5 standard deviations.

### Methylation over orthologous gene bodies

Gene annotations for each species as well orthologous gene information were obtained from Ensembl's biomart. Promoter and CpG island sequences were removed from each gene before calculating average percent methylation over gene bodies. Genes with fewer than 20 remaining CpG sites with methylation information were removed from each species' dataset.

## Supporting Information

S1 Fig Methylation levels in placenta promoters, CpG islands, and gene bodies. Violin plots of percent methylation, with the median shown as a white dot and the interquartile range shown as a black bar. Gene annotations were taken from Ensembl. Promoters were defined as 1000 bp upstream and 100 bp downstream of the transcription start site. CpG islands and promoters had to have at least 10 CpG sites with methylation data. Genes had to have at least 20 CpG sites with methylation data to be included. Gene annotation data for squirrel monkey was not available on Ensembl.
(TIFF)

S2 Fig Methylation levels in repetitive elements. Using RepeatMasker annotations for each species, average methylation was calculated for all CpG sites in LINEs, SINEs, and LTRs. Average methylation in non-repetitive sequences (CpG sites outside all known repetitive sequences) is also shown. In the placentas of all species, LINEs and LTRs have similar or slightly higher methylation levels compared to non-repetitive sequences. SINEs have significantly higher methylation, but absolute methylation levels in SINEs still follow that of the non-repetitive sequence in the placenta and do not reach the 85% methylation levels seen in the adult brain. EEM = extraembryonic membrane.
(TIFF)

S3 Fig Influence of window length on PMD/HMD detection in mammalian placentas. Density curves of percent methylation in non-overlapping windows of various lengths. With 2 kb window lengths, CpG islands can be seen as a bump near 0% methylation. In human placenta a bimodal distribution can be seen with window lengths of 5–50 kb. With windows lengths above 50 kb, regions of low and high methylation are combined and the bimodal distribution is lost. The dog placenta methylome shows a similar bimodal distribution. Mouse, horse, and cow placentas, however, show little evidence for bimodal distributions regardless of the window length. Rhesus has a small secondary peak at higher methylation levels that is best seen with 20 kb window lengths.
(TIFF)

S4 Fig Comparison of global methylation patterns in select species after liftOver to the human genome and smoothing. The first 120 Mb of human chromosome 1 are shown. Human placenta PMDs, as determined by HMM, are shown in black bars at top. Breaks in large regions of chromosomal synteny between human and the comparison species are shown as vertical gray lines. Due to the relatively long evolutionary time since the last common ancestor of human and opossum, the liftOver of opossum CpG sites to the human genome was largely unsuccessful. Artifacts can be seen in the mouse and opossum methylation curves (levels spiking below 0% or above 100%) where smoothing was done over genomic regions with no methylation coverage due to unsuccessful liftOvers in that region.
(TIFF)

S5 Fig Comparison of methylation patterns at the *CNTNAP2* locus. Raw species CpG site percent methylation wig data were graphed on the UCSC Genome Browser without preprocessing. Genes of interest are colored in orange. Black lines represent 50% methylation. In squirrel monkey the end of contig JH378140 occurs within *DPP6*. For visual clarity the small fragment from the other contig (containing *PAXIP1* and *RBM33*) was not diagrammed. The mouse genome underwent an interchromosomal break in the middle of this locus and is split between chromosomes 5 and 6. The dog genome underwent an intrachromosomal rearrangement. In the interest of diagramming the entire locus intact, the length of the genome shown for dog is much longer than that for the other species. Note that some genes such as *EZH2*, *KMT2C*, *PAXIP1*, and *RBM33* that have relatively high methylation in human tend to be highly methylated in all species (squirrel monkey as well, not all data shown). Long human PMDs (such as those over *CNTNAP2* and *DPP6*) are conserved in some species, but in other species these patterns are not as striking or are entirely absent.
(TIFF)

S6 Fig Comparison of methylation patterns at the *DAB1* locus. Raw species CpG site percent methylation wig data were graphed on the UCSC Genome Browser without preprocessing. Genes of interest are colored in orange. Black lines represent 50% methylation. The mouse genome underwent a local rearrangement at this locus. The horse genome underwent an interchromosomal break in the middle of this locus and is split between chromosomes 2 and 5. Note that some genes such as *USP24*, *FGGY*, *INADL*, *DOCK7*, *ATG4A*, and *ALG6* that have relatively high methylation in human tend to be highly methylated in most if not all species. Long human PMDs (such as the one over *DAB1*) are conserved in some species, but in other species these patterns are not as striking or are entirely absent.
(TIFF)

S7 Fig Reproducibility of rhesus and mouse placenta MethylC-seq data. (A-C) Reproducibility of methylation patterns in whole rhesus placenta and isolated rhesus trophoblast cells. (D-F) Reproducibility of methylation patterns in whole mouse placenta across labs. MethylC-seq data from our lab using E15.5 and E11.5 placentas has virtually identical patterns compared to MethylC-seq data from Hon et al. (2013) (GSE42836) using E15.5 placenta. All samples were from C57Bl/6 mice. (A, D) Methylation patterns across the first 120 Mb of each species' chromosome 1. Methylation data were compressed into averages of non-overlapping 20 kb windows (windows with less than 20 CpG sites with methylation information were discarded) and smoothed in R using a kernel smoother. (B, E) Density curves of average percent methylation in non-overlapping 20 kb windows. (C, F) Pearson correlations between the average methylation levels in 20 kb windows. The red line designates the path of a perfect correlation.
(TIFF)

S8 Fig Reproducibility of cow and dog placenta MethylC-seq data. (A-C) Reproducibility of methylation patterns in four whole cow placenta biological replicates. Placentas 2–4 came from a different source than placenta 1. (D-F) Reproducibility of methylation patterns in three whole dog placentas across different developmental timepoints and breeds. Placenta 1 is from a Vizsla, gestation day 40–45. Placenta 2 is from a pit bull, gestation day 21–25. Placenta 3 is from a terrier, gestation day 30–35. (A, D) Methylation patterns across the first 120 Mb of each species' chromosome 1. Methylation data were compressed into averages of non-overlapping 20 kb windows (windows with less than 20 CpG sites with methylation information were discarded) and smoothed in R using a kernel smoother. (B, E) Density curves of average percent methylation in non-overlapping 20 kb windows. (C, F) Pearson correlation between the average methylation levels in 20 kb windows. The red line designates the path of a perfect correlation.
(TIFF)

S9 Fig Reproducibility of human placenta MethylC-seq data. (A-C) Reproducibility of methylation patterns in human placenta across labs and sequencing coverage. MethylC-seq data for three human placenta samples from our lab (GSE39775) have virtually identical patterns compared to MethylC-seq data from Court et al. (2014) (GSE46698). (A) Methylation patterns across the first 120 Mb of chromosome 1. Methylation data were compressed into averages of non-overlapping 20 kb windows (windows with less than 20 CpG sites with methylation information were discarded) and smoothed in R using a kernel smoother. The three samples from Schroeder et al. (2013) are in shades of red. The sample from Court et al. is in blue. (B) Density curves of average percent methylation in non-overlapping 20 kb windows. (C) Pearson correlations between the average methylation levels in 20 kb windows. The red line designates the path of a perfect correlation.
(TIFF)

S10 Fig Relationship between methylation and genic/chromosomal features. A) Graph of methylation differences and large chromosomal syntenic breaks across mammalian placentas. Species placenta methylation data were lifted over to the human genome using liftOver. Bars within the colored rings show large chromosomal breaks in synteny compared to human: purple = rhesus, orange = squirrel monkey, gray = mouse, blue = dog, yellow = horse, and green = cow. Above each colored ring are the color-coded differences in methylation between that species' placenta and human placenta. Regions of higher methylation in human placenta are in blue, regions of lower methylation in red. For comparison, the inner-most circle of differential methylation is between human placenta and human cord blood. (B) Distance of human placenta boundaries to gene ends and CpG islands compared to distances expected by chance.
(TIFF)

S11 Fig Distribution of methylation inside and outside of genes in placentas and brains. Non-overlapping 5 kb windows were tiled across the autosomes and those with a minimum of 10 covered CpG sites were used. Windows were classified as being genic or intergenic and the methylation distribution was plotted for each. Artifacts can be seen at the 100% methylation level in the human brain data due to low sequencing coverage.
(TIFF)

S12 Fig Value of percent methylation in predicting gene location in placentas and brains. Same data as in S11 Fig, this time showing spinograms of the probability that a 5 kb window is in a gene given that window's average percent methylation. Bars are color-coded by percent methylation and bar widths show the percentage of windows with that methylation level. Bars furthest from the 0.5 blue line show the most information about gene location.
(TIFF)

S13 Fig Global and gene body methylation distributions during early mouse, human, and cow development. Mouse gamete and early embryo data are from Wang et al. (2014) (GSE56697). Human oocyte and sperm data are from Okae et al. (2014) (JGAS00000000006). Human ICM data are from Guo et al (2014) (GSE49828). Note the bimodal peaks in gene body methylation in the oocytes of all species as well as mouse 2-cell through ICM preimplantation embryos.
(TIFF)

S14 Fig Methylation correlations during early human development. Correlation of (A) average methylation in 50 kb windows and (B) average gene body methylation during early human development. Human oocyte, sperm, and blastocyst data are from Okae et al. (2014) (JGAS00000000006). Human ICM data are from Guo et al. (2014) (GSE49828). Gray and purple show regions of low and high density, respectively.
(TIFF)

S15 Fig Gene body methylation during early mouse development. Correlation of average gene body methylation in individual genes across mouse developmental timepoints. Data from Wang et al. (2014) (GSE56697). Gray and purple show regions of low and high density, respectively.
(TIFF)

S16 Fig Value of percent methylation in predicting gene location in human, mouse, and cow oocytes and during early mouse development. Data were processed and graphed as in S11 and S12 Figs.
(TIFF)

S17 Fig Functions of genes differentially expressed in mouse oocytes and placenta. (A-B) Gene expression in (A) human and (B) mouse placenta versus oocytes is shown, divided into quadrants based on high/low expression in each tissue. Both x and y axes show log(FPKM)+1, with expression thresholds set to 0.56. For the genes in each quadrant, the top few GO biological process terms and Benjamini p-values are listed. For a complete list see S3 Table. Note that genes with high transcript levels in both human and mouse oocytes and placenta are enriched for many of the same functions as the genes with conserved high methylation across mammalian placentas (Fig 2A). (C) Venn diagram showing overlap of genes with high gene body methylation in mammalian placentas (see Fig 2A), high gene body methylation in human, mouse, and cow oocytes (see Fig 4B), and high expression in human and mouse placenta and oocytes (see A-B above). Mouse numbers are shown in parentheses. Note that the first two gene body methylation datasets only used Ensembl genes with orthologs across multiple mammalian species. The gene expression datasets used all RefSeq human and mouse genes.
(TIFF)

S1 Table Information about species, tissue samples, and MethylC-seq statistics. (XLS)

S2 Table Methylation statistics for 20 kb windows in mammalian placentas. Note that biases can occur in the cross-species correlation statistic for species such as opossum due to the relatively small percentage of the genome can be mapped to human.
(XLS)

S3 Table GO term and KEGG pathway enrichment. Only GO biological process terms and KEGG pathways with Benjamini p-values below 1.0E-2 are shown.
(XLS)
