# Supplementary figures and images for: Early Developmental and Evolutionary Origins of Gene Body DNA Methylation Patterns in Mammalian Placentas
Source: PLoS Genet. 2015 Aug 4;11(8):e1005442. doi: 10.1371/journal.pgen.1005442 (PMC4524645; doi:10.1371/journal.pgen.1005442)

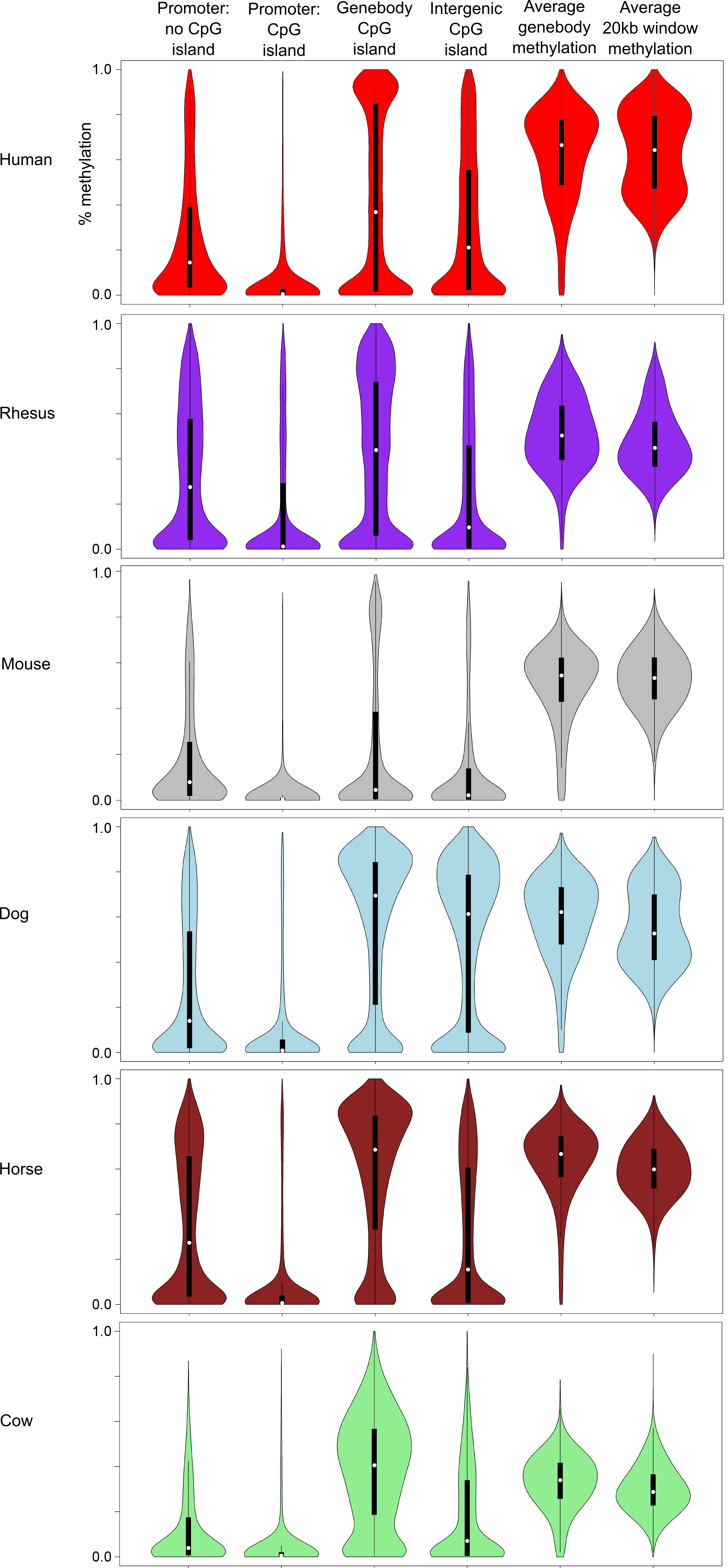

Supplement: S1 Fig — Violin plots of percent methylation, with the median shown as a white dot and the interquartile range shown as a black bar. Gene annotations were taken from Ensembl. Promoters were defined as 1000 bp upstream and 100 bp downstream of the transcription start site. CpG islands and promoters had to have at least 10 CpG sites with methylation data. Genes had to have at least 20 CpG sites with methylation data to be included. Gene annotation data for squirrel monkey was not available on Ensembl. (TIFF) [file pgen.1005442.s001.tiff]

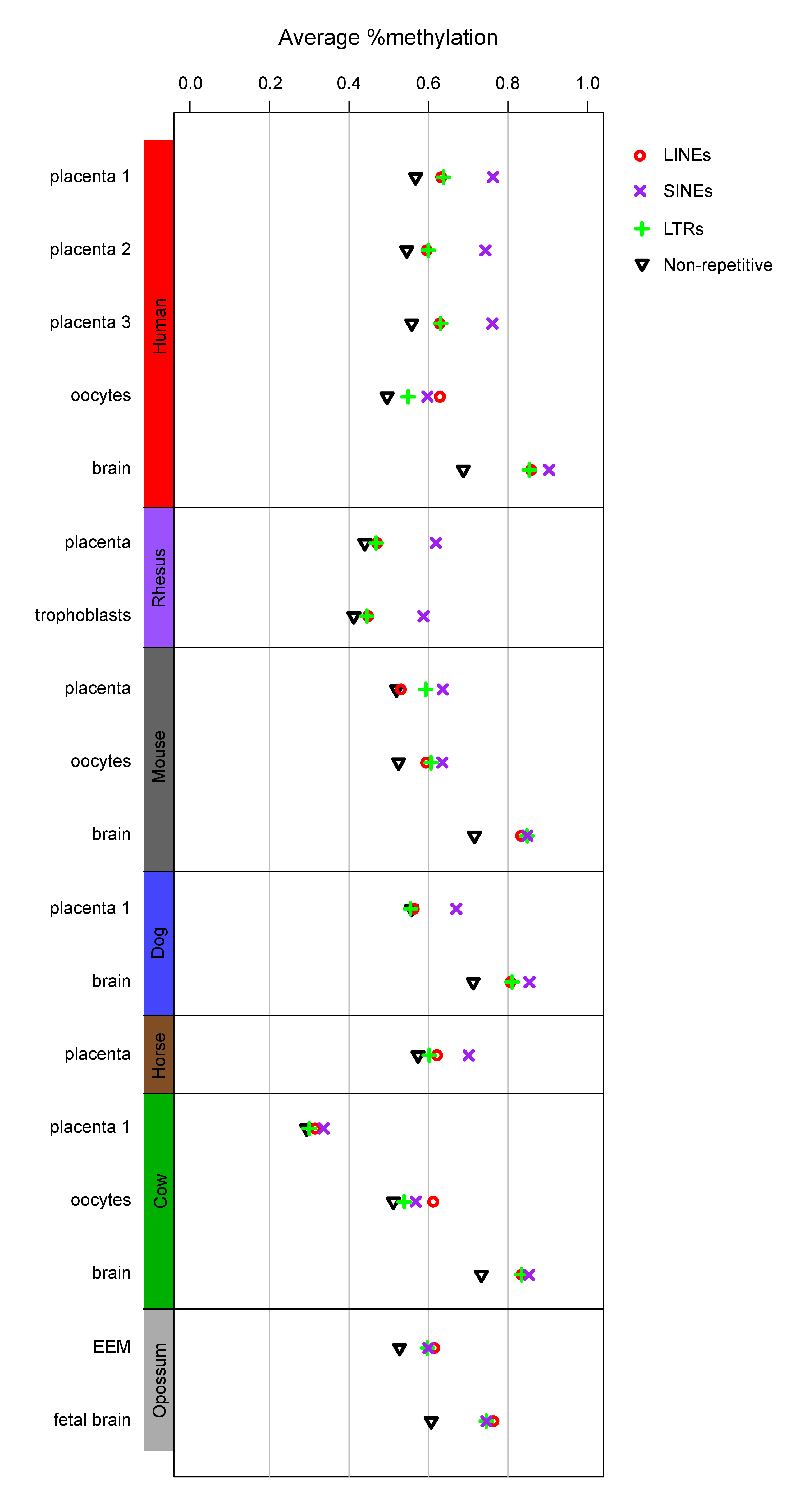

Supplement: S2 Fig — Using RepeatMasker annotations for each species, average methylation was calculated for all CpG sites in LINEs, SINEs, and LTRs. Average methylation in non-repetitive sequences (CpG sites outside all known repetitive sequences) is also shown. In the placentas of all species, LINEs and LTRs have similar or slightly higher methylation levels compared to non-repetitive sequences. SINEs have significantly higher methylation, but absolute methylation levels in SINEs still follow that of the non-repetitive sequence in the placenta and do not reach the 85% methylation levels seen in the adult brain. EEM = extraembryonic membrane. (TIFF) [file pgen.1005442.s002.tiff]

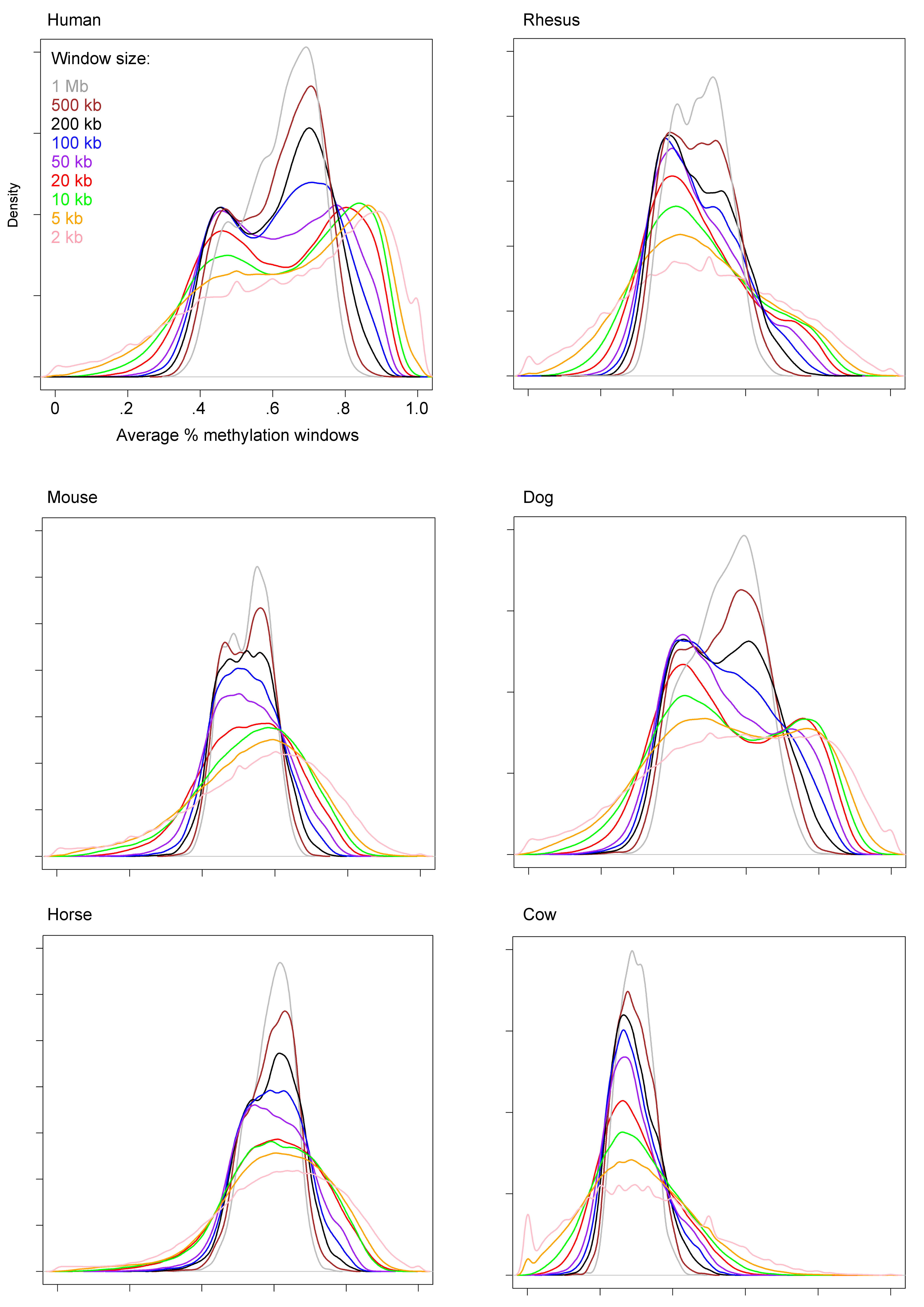

Supplement: S3 Fig — Density curves of percent methylation in non-overlapping windows of various lengths. With 2 kb window lengths, CpG islands can be seen as a bump near 0% methylation. In human placenta a bimodal distribution can be seen with window lengths of 5–50 kb. With windows lengths above 50 kb, regions of low and high methylation are combined and the bimodal distribution is lost. The dog placenta methylome shows a similar bimodal distribution. Mouse, horse, and cow placentas, however, show little evidence for bimodal distributions regardless of the window length. Rhesus has a small secondary peak at higher methylation levels that is best seen with 20 kb window lengths. (TIFF) [file pgen.1005442.s003.tiff]

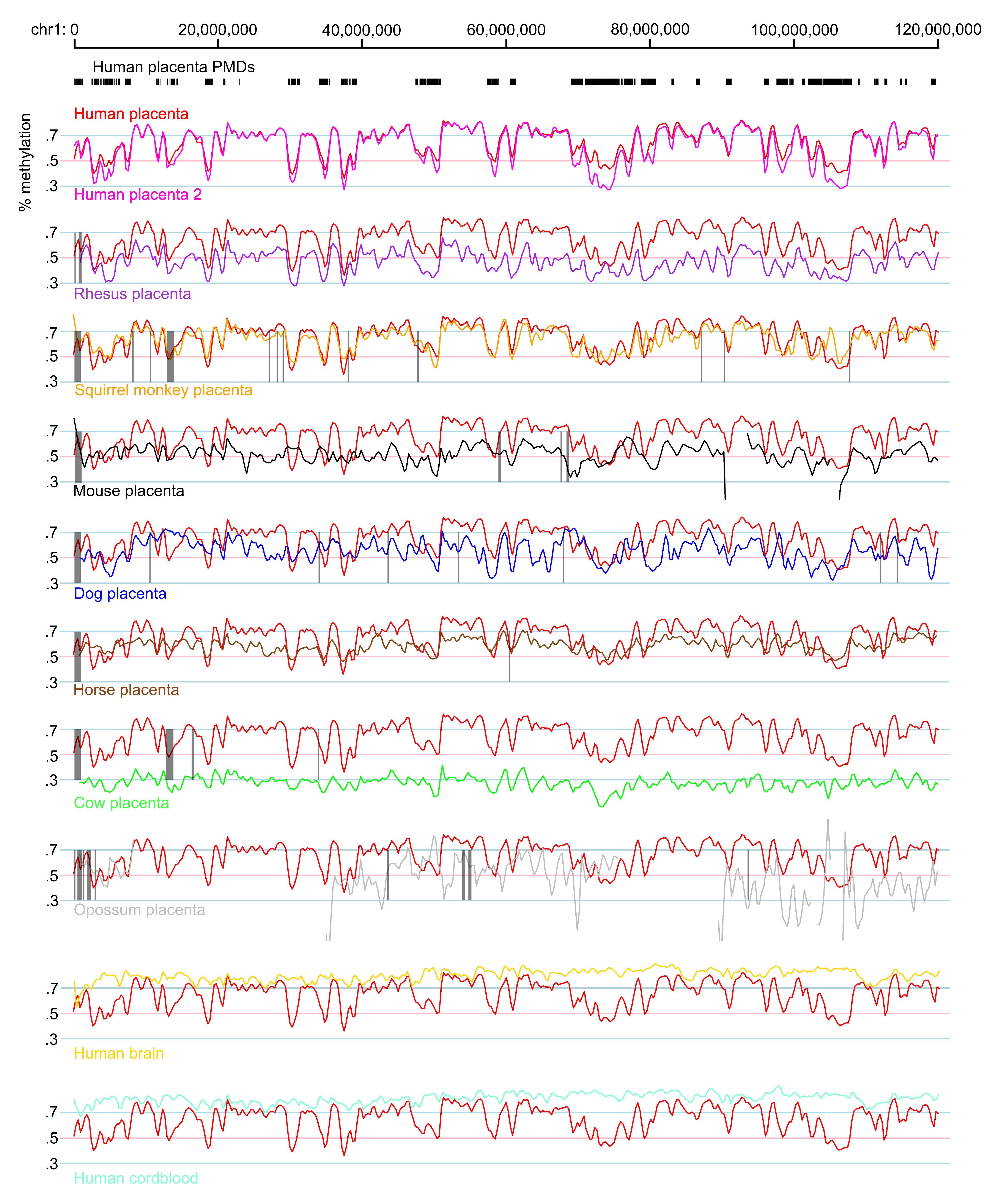

Supplement: S4 Fig — The first 120 Mb of human chromosome 1 are shown. Human placenta PMDs, as determined by HMM, are shown in black bars at top. Breaks in large regions of chromosomal synteny between human and the comparison species are shown as vertical gray lines. Due to the relatively long evolutionary time since the last common ancestor of human and opossum, the liftOver of opossum CpG sites to the human genome was largely unsuccessful. Artifacts can be seen in the mouse and opossum methylation curves (levels spiking below 0% or above 100%) where smoothing was done over genomic regions with no methylation coverage due to unsuccessful liftOvers in that region. (TIFF) [file pgen.1005442.s004.tiff]

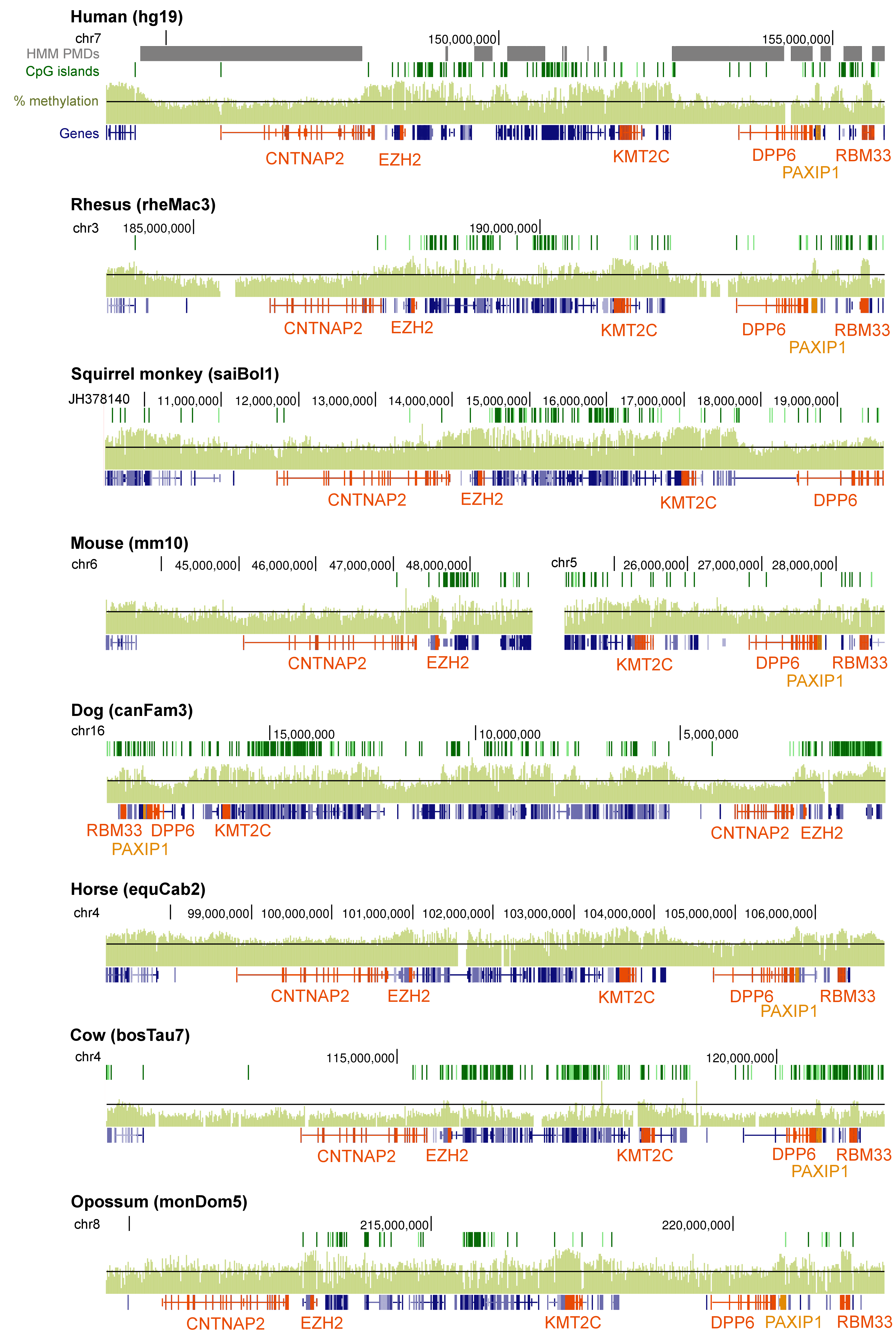

Supplement: S5 Fig — Raw species CpG site percent methylation wig data were graphed on the UCSC Genome Browser without preprocessing. Genes of interest are colored in orange. Black lines represent 50% methylation. In squirrel monkey the end of contig JH378140 occurs within DPP6. For visual clarity the small fragment from the other contig (containing PAXIP1 and RBM33) was not diagrammed. The mouse genome underwent an interchromosomal break in the middle of this locus and is split between chromosomes 5 and 6. The dog genome underwent an intrachromosomal rearrangement. In the interest of diagramming the entire locus intact, the length of the genome shown for dog is much longer than that for the other species. Note that some genes such as EZH2, KMT2C, PAXIP1, and RBM33 that have relatively high methylation in human tend to be highly methylated in all species (squirrel monkey as well, not all data shown). Long human PMDs (such as those over CNTNAP2 and DPP6) are conserved in some species, but in other species these patterns are not as striking or are entirely absent. (TIFF) [file pgen.1005442.s005.tiff]

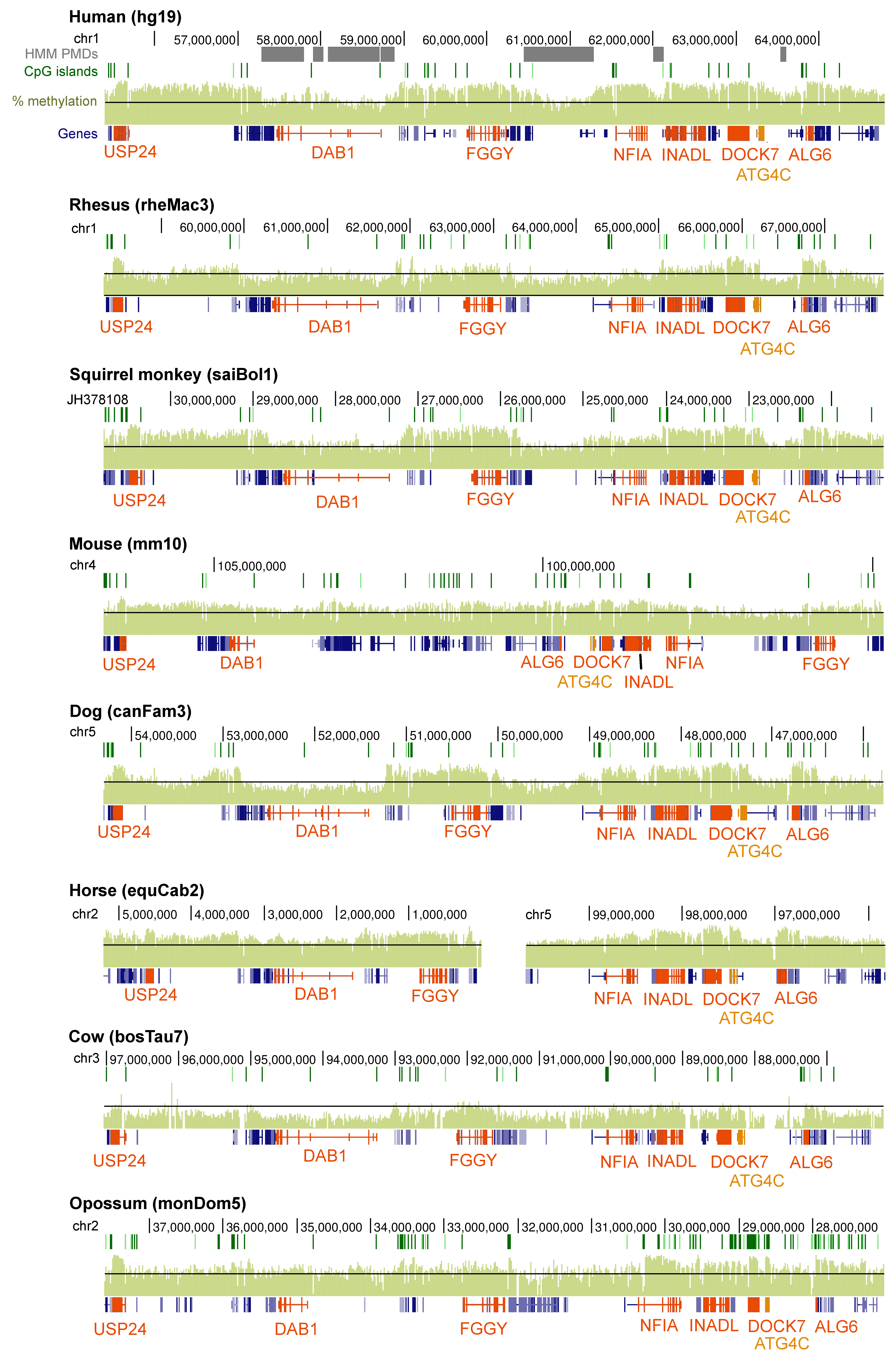

Supplement: S6 Fig — Raw species CpG site percent methylation wig data were graphed on the UCSC Genome Browser without preprocessing. Genes of interest are colored in orange. Black lines represent 50% methylation. The mouse genome underwent a local rearrangement at this locus. The horse genome underwent an interchromosomal break in the middle of this locus and is split between chromosomes 2 and 5. Note that some genes such as USP24, FGGY, INADL, DOCK7, ATG4A, and ALG6 that have relatively high methylation in human tend to be highly methylated in most if not all species. Long human PMDs (such as the one over DAB1) are conserved in some species, but in other species these patterns are not as striking or are entirely absent. (TIFF) [file pgen.1005442.s006.tiff]

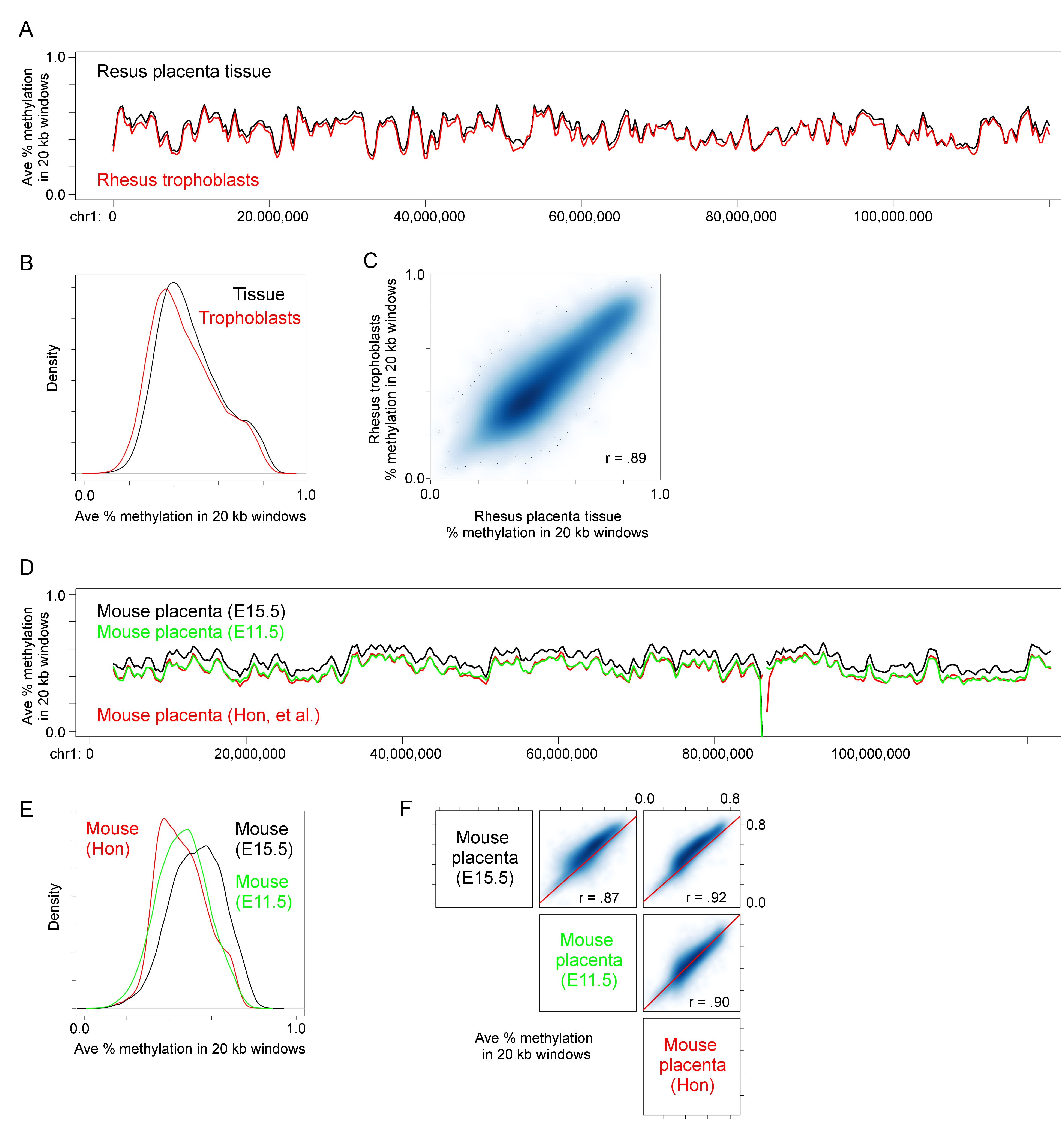

Supplement: S7 Fig — (A-C) Reproducibility of methylation patterns in whole rhesus placenta and isolated rhesus trophoblast cells. (D-F) Reproducibility of methylation patterns in whole mouse placenta across labs. MethylC-seq data from our lab using E15.5 and E11.5 placentas has virtually identical patterns compared to MethylC-seq data from Hon et al. (2013) (GSE42836) using E15.5 placenta. All samples were from C57Bl/6 mice. (A, D) Methylation patterns across the first 120 Mb of each species' chromosome 1. Methylation data were compressed into averages of non-overlapping 20 kb windows (windows with less than 20 CpG sites with methylation information were discarded) and smoothed in R using a kernel smoother. (B, E) Density curves of average percent methylation in non-overlapping 20 kb windows. (C, F) Pearson correlations between the average methylation levels in 20 kb windows. The red line designates the path of a perfect correlation. (TIFF) [file pgen.1005442.s007.tiff]

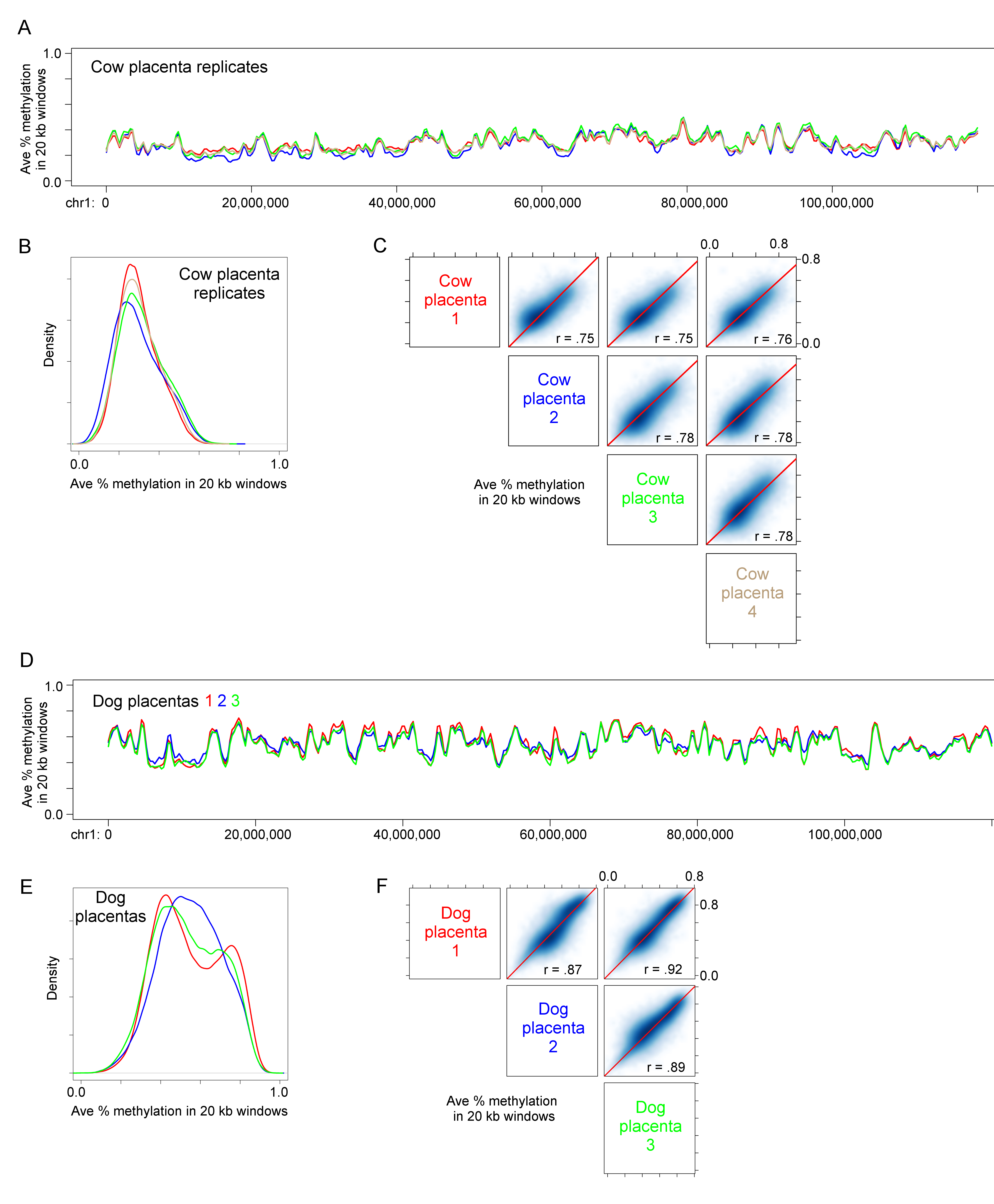

Supplement: S8 Fig — (A-C) Reproducibility of methylation patterns in four whole cow placenta biological replicates. Placentas 2–4 came from a different source than placenta 1. (D-F) Reproducibility of methylation patterns in three whole dog placentas across different developmental timepoints and breeds. Placenta 1 is from a Vizsla, gestation day 40–45. Placenta 2 is from a pit bull, gestation day 21–25. Placenta 3 is from a terrier, gestation day 30–35. (A, D) Methylation patterns across the first 120 Mb of each species' chromosome 1. Methylation data were compressed into averages of non-overlapping 20 kb windows (windows with less than 20 CpG sites with methylation information were discarded) and smoothed in R using a kernel smoother. (B, E) Density curves of average percent methylation in non-overlapping 20 kb windows. (C, F) Pearson correlation between the average methylation levels in 20 kb windows. The red line designates the path of a perfect correlation. (TIFF) [file pgen.1005442.s008.tiff]

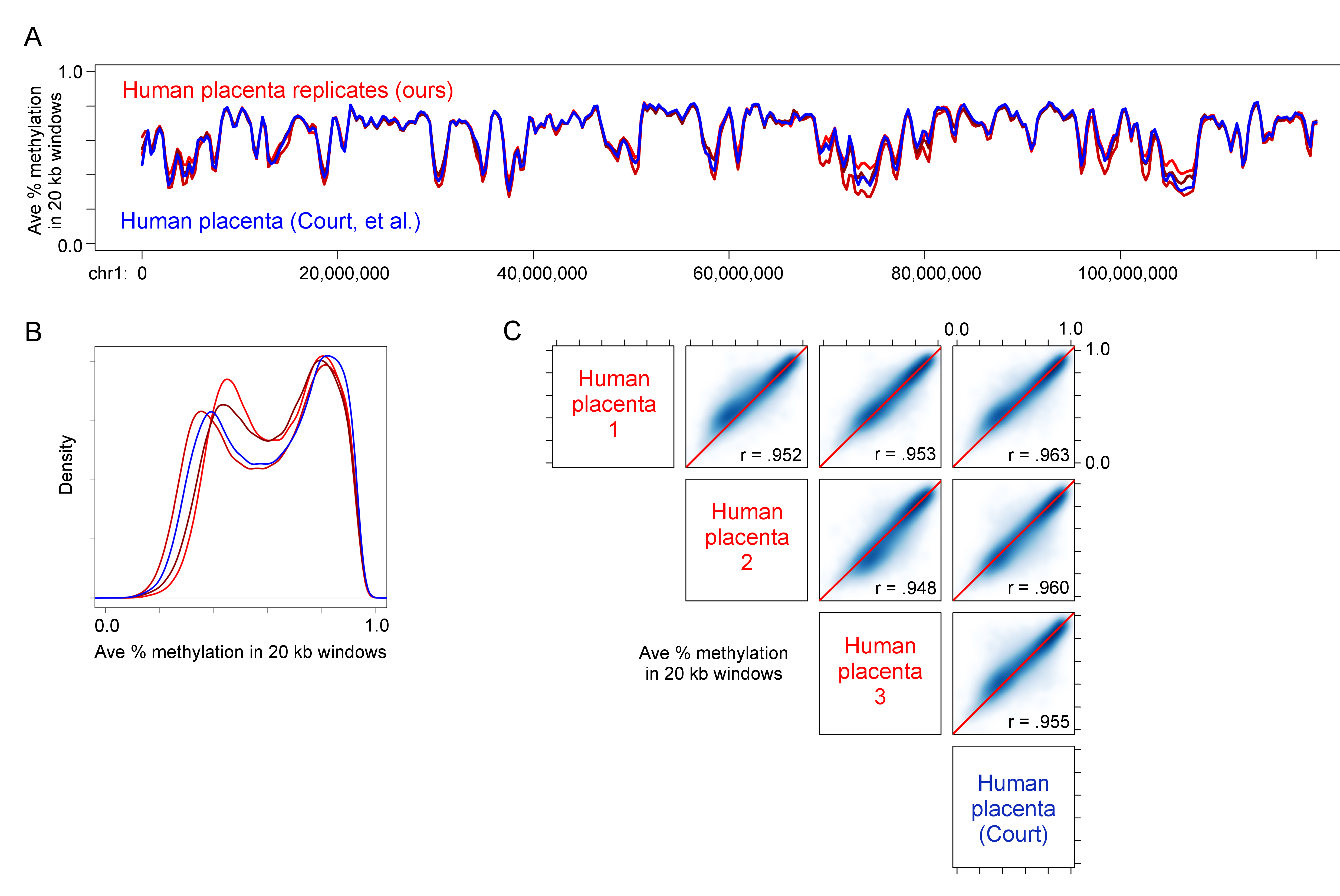

Supplement: S9 Fig — (A-C) Reproducibility of methylation patterns in human placenta across labs and sequencing coverage. MethylC-seq data for three human placenta samples from our lab (GSE39775) have virtually identical patterns compared to MethylC-seq data from Court et al. (2014) (GSE46698). (A) Methylation patterns across the first 120 Mb of chromosome 1. Methylation data were compressed into averages of non-overlapping 20 kb windows (windows with less than 20 CpG sites with methylation information were discarded) and smoothed in R using a kernel smoother. The three samples from Schroeder et al. (2013) are in shades of red. The sample from Court et al. is in blue. (B) Density curves of average percent methylation in non-overlapping 20 kb windows. (C) Pearson correlations between the average methylation levels in 20 kb windows. The red line designates the path of a perfect correlation. (TIFF) [file pgen.1005442.s009.tiff]

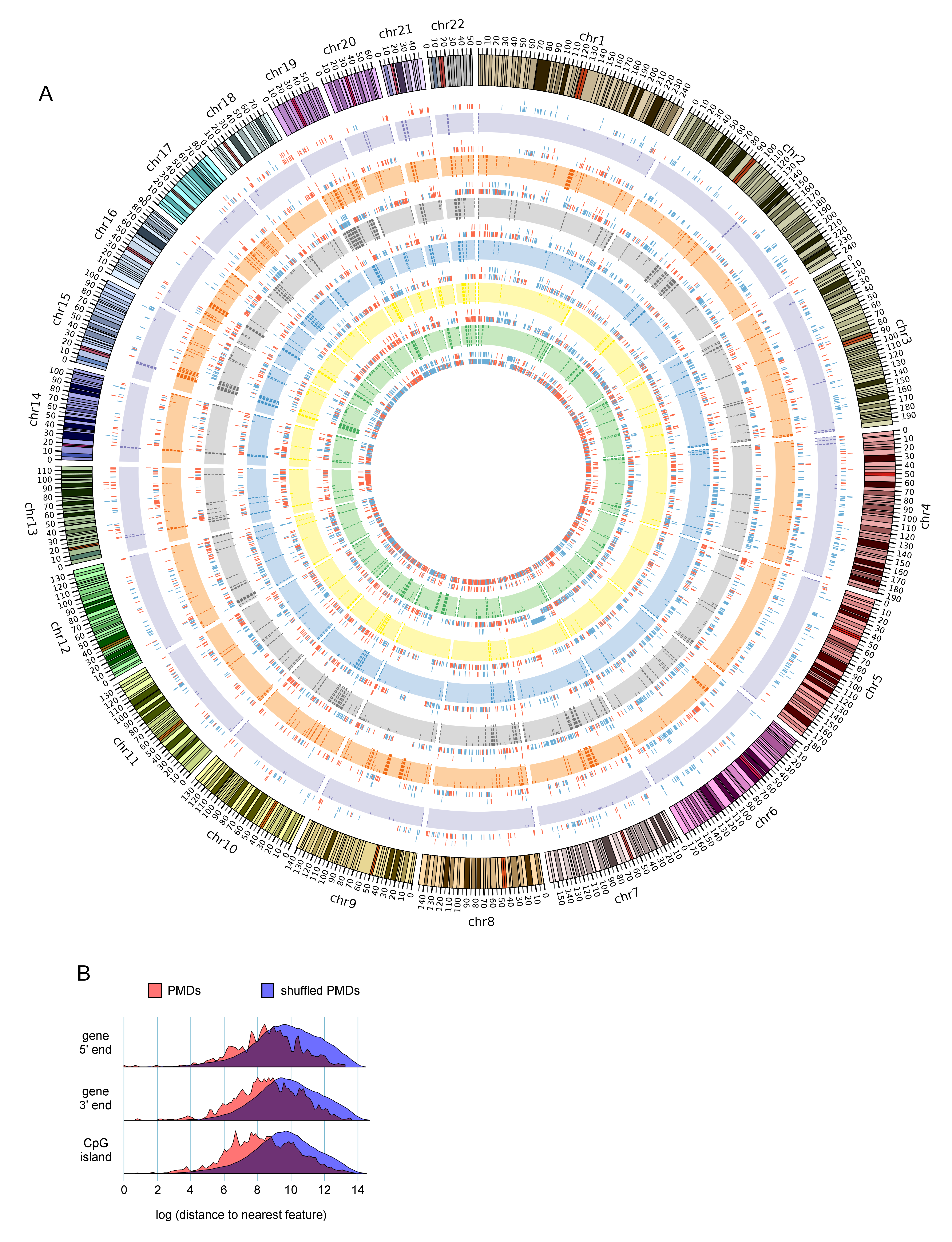

Supplement: S10 Fig — A) Graph of methylation differences and large chromosomal syntenic breaks across mammalian placentas. Species placenta methylation data were lifted over to the human genome using liftOver. Bars within the colored rings show large chromosomal breaks in synteny compared to human: purple = rhesus, orange = squirrel monkey, gray = mouse, blue = dog, yellow = horse, and green = cow. Above each colored ring are the color-coded differences in methylation between that species' placenta and human placenta. Regions of higher methylation in human placenta are in blue, regions of lower methylation in red. For comparison, the inner-most circle of differential methylation is between human placenta and human cord blood. (B) Distance of human placenta boundaries to gene ends and CpG islands compared to distances expected by chance. (TIFF) [file pgen.1005442.s010.tiff]

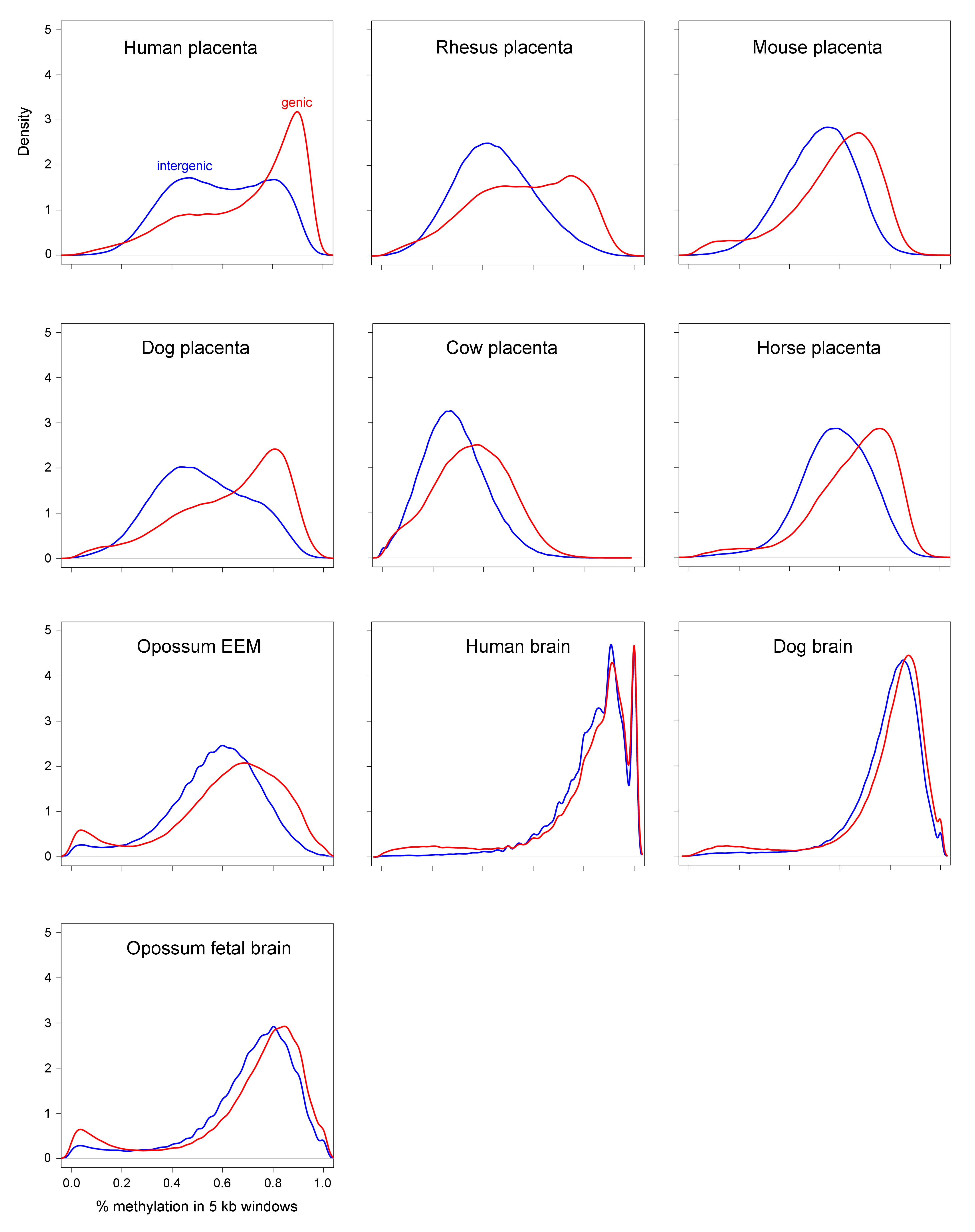

Supplement: S11 Fig — Non-overlapping 5 kb windows were tiled across the autosomes and those with a minimum of 10 covered CpG sites were used. Windows were classified as being genic or intergenic and the methylation distribution was plotted for each. Artifacts can be seen at the 100% methylation level in the human brain data due to low sequencing coverage. (TIFF) [file pgen.1005442.s011.tiff]

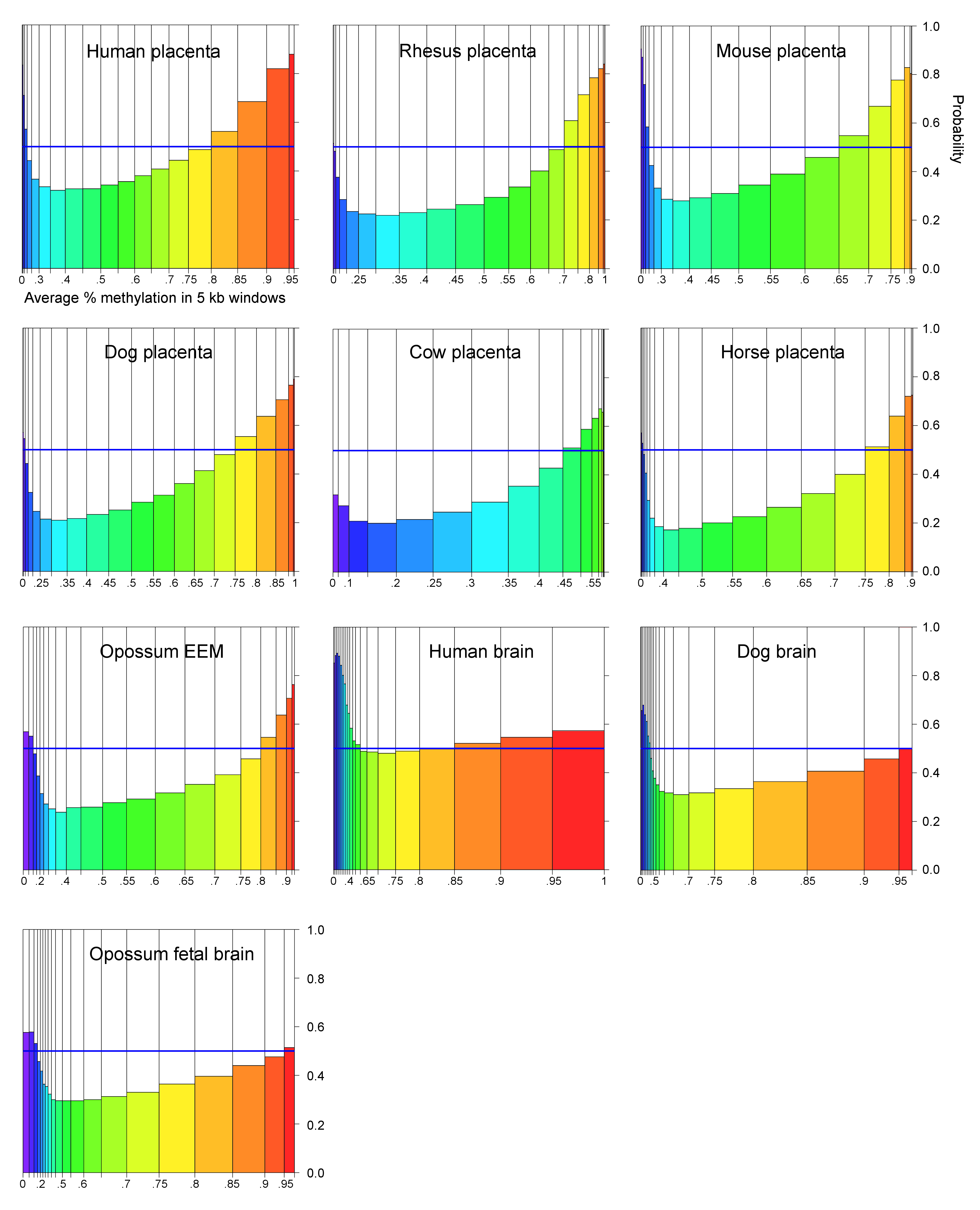

Supplement: S12 Fig — Same data as in S11 Fig, this time showing spinograms of the probability that a 5 kb window is in a gene given that window's average percent methylation. Bars are color-coded by percent methylation and bar widths show the percentage of windows with that methylation level. Bars furthest from the 0.5 blue line show the most information about gene location. (TIFF) [file pgen.1005442.s012.tiff]

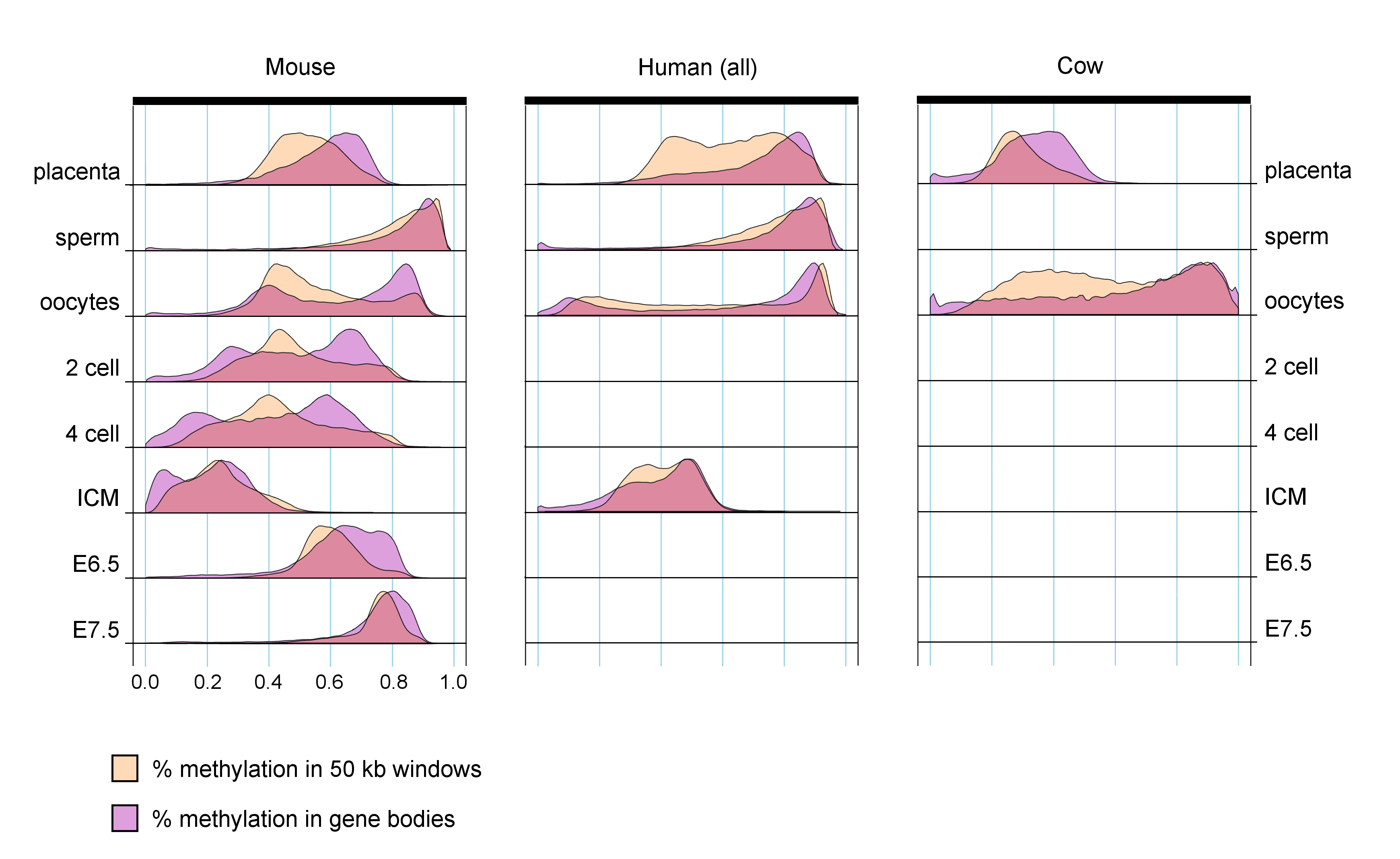

Supplement: S13 Fig — Mouse gamete and early embryo data are from Wang et al. (2014) (GSE56697). Human oocyte and sperm data are from Okae et al. (2014) (JGAS00000000006). Human ICM data are from Guo et al (2014) (GSE49828). Note the bimodal peaks in gene body methylation in the oocytes of all species as well as mouse 2-cell through ICM preimplantation embryos. (TIFF) [file pgen.1005442.s013.tiff]

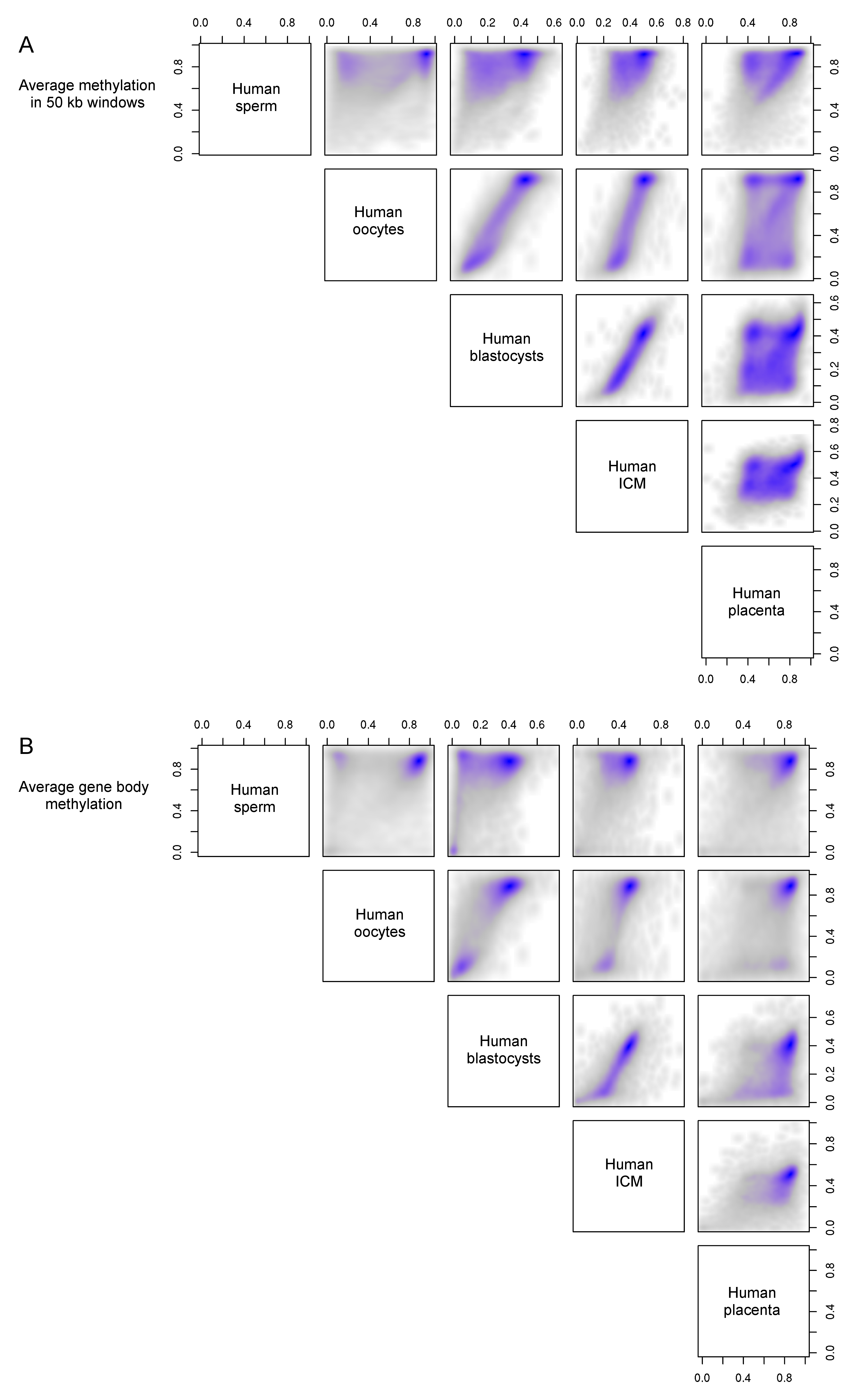

Supplement: S14 Fig — Correlation of (A) average methylation in 50 kb windows and (B) average gene body methylation during early human development. Human oocyte, sperm, and blastocyst data are from Okae et al. (2014) (JGAS00000000006). Human ICM data are from Guo et al. (2014) (GSE49828). Gray and purple show regions of low and high density, respectively. (TIFF) [file pgen.1005442.s014.tiff]

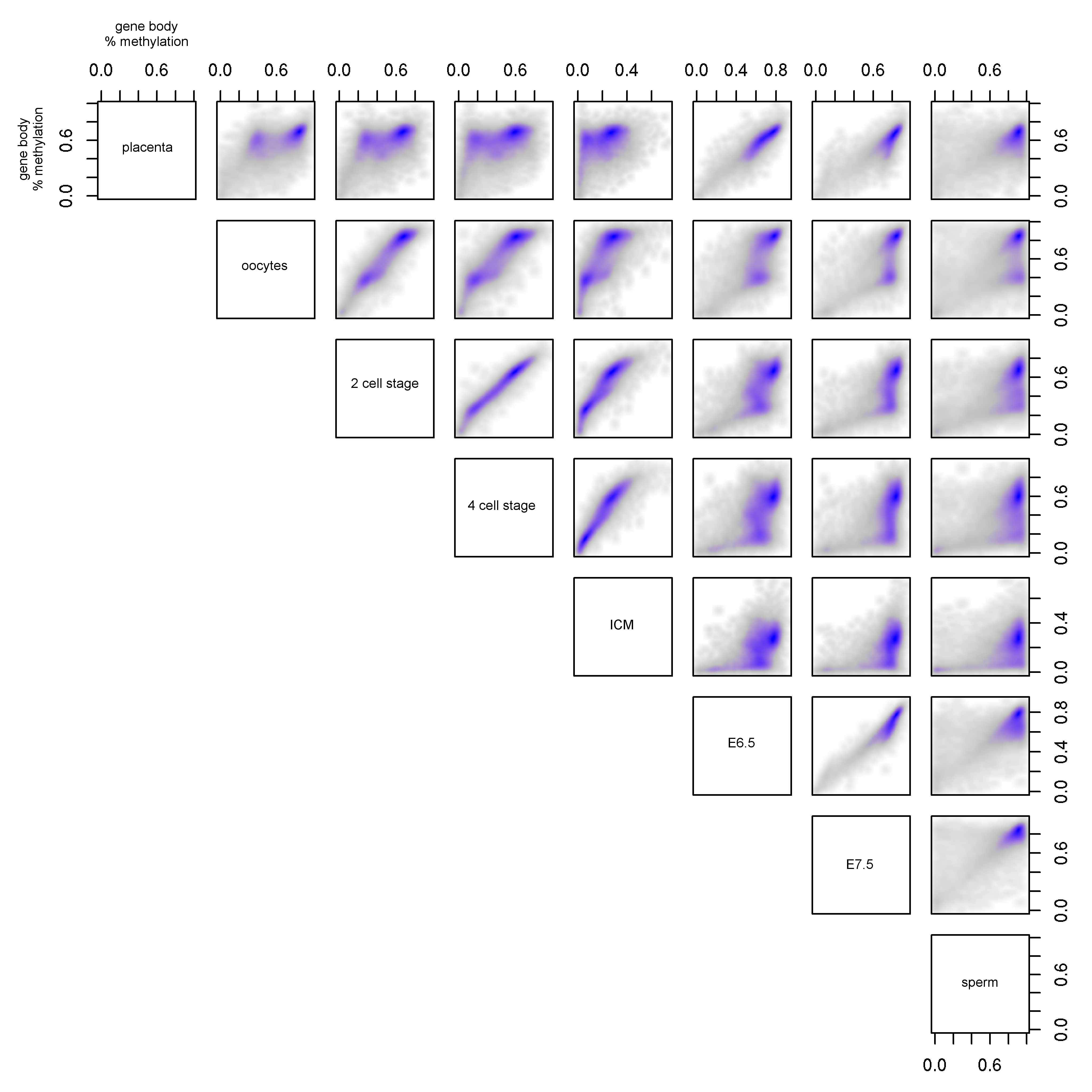

Supplement: S15 Fig — Correlation of average gene body methylation in individual genes across mouse developmental timepoints. Data from Wang et al. (2014) (GSE56697). Gray and purple show regions of low and high density, respectively. (TIFF) [file pgen.1005442.s015.tiff]

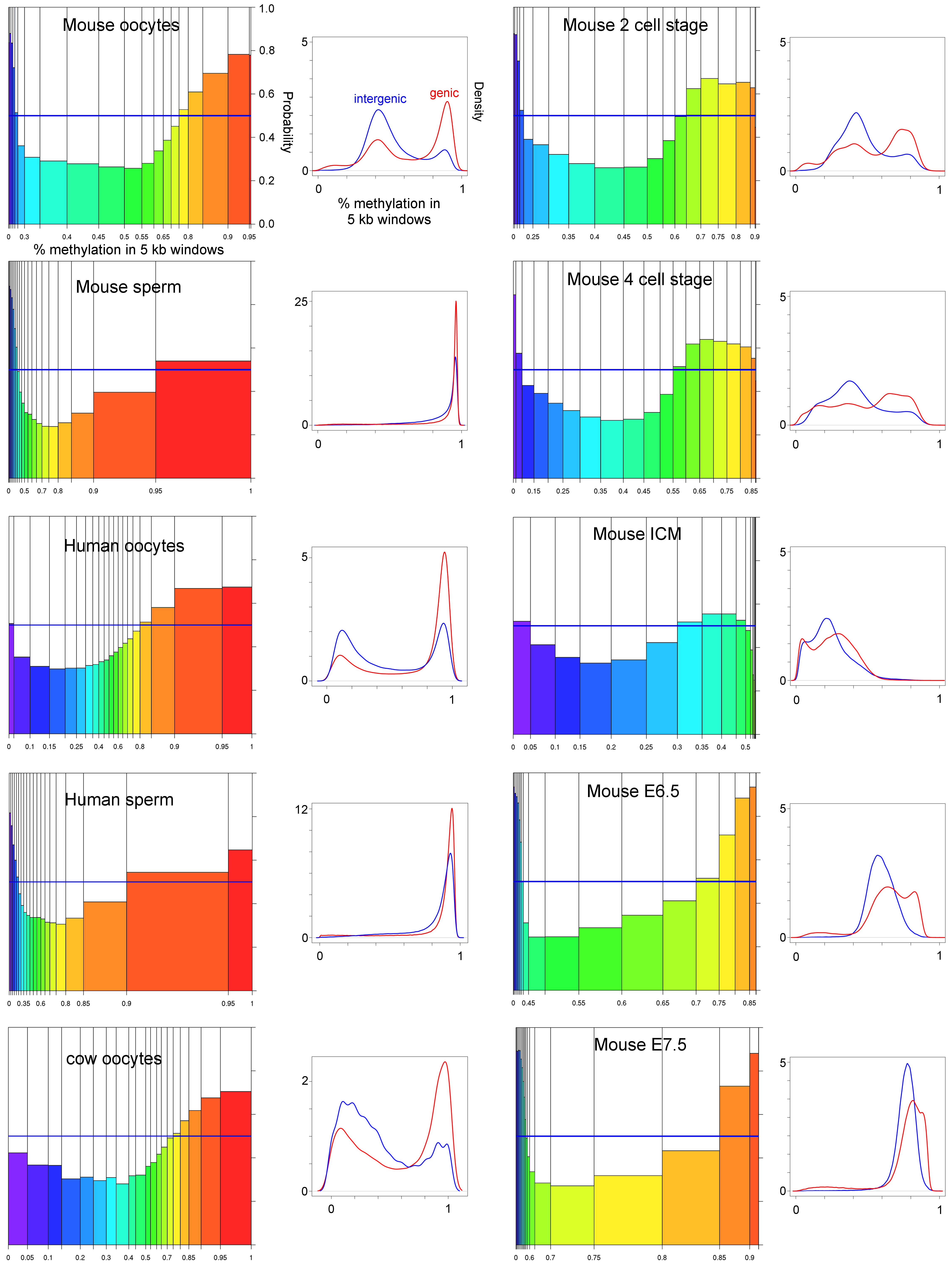

Supplement: S16 Fig — Data were processed and graphed as in S11 and S12 Figs. (TIFF) [file pgen.1005442.s016.tiff]

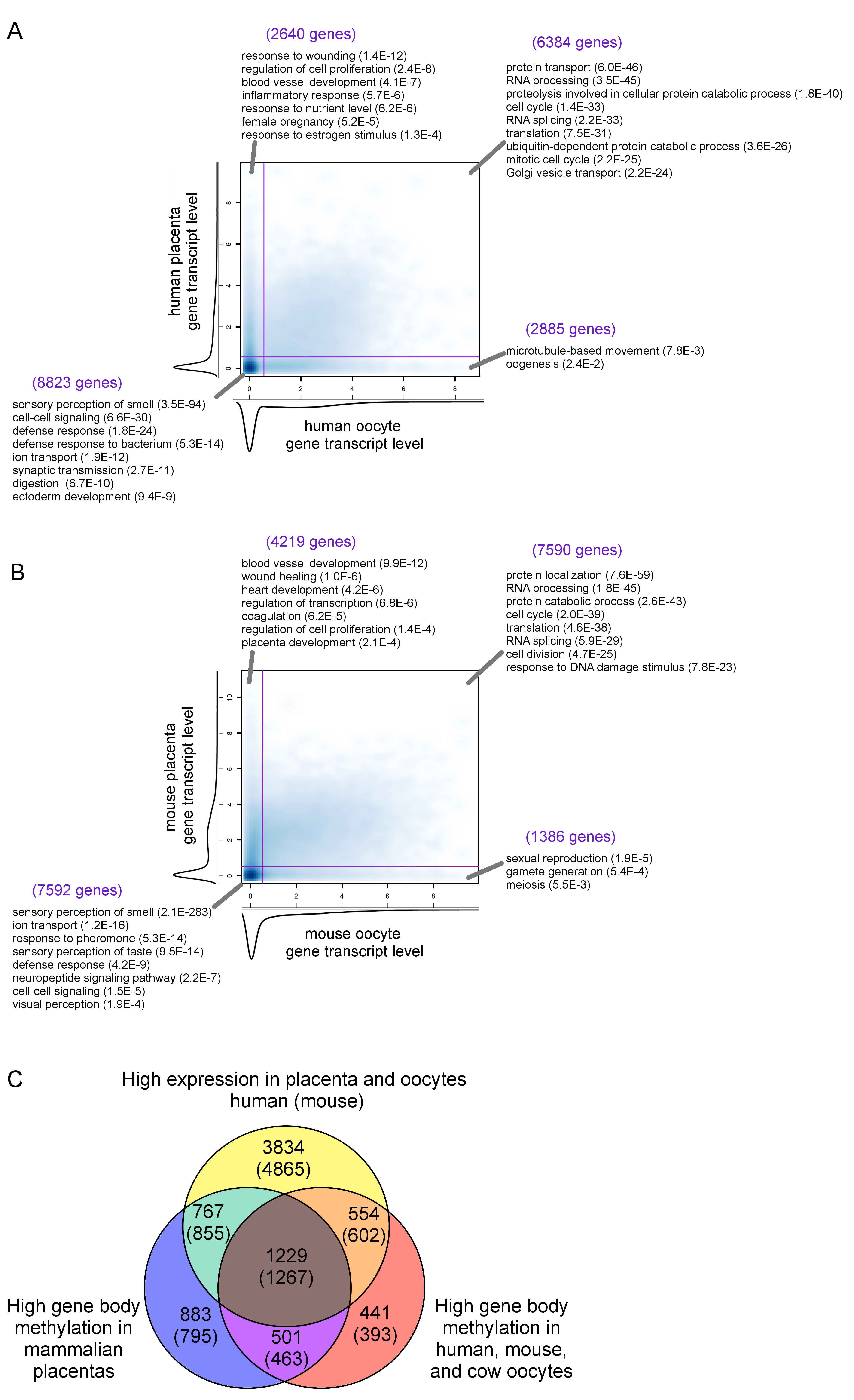

Supplement: S17 Fig — (A-B) Gene expression in (A) human and (B) mouse placenta versus oocytes is shown, divided into quadrants based on high/low expression in each tissue. Both x and y axes show log(FPKM)+1, with expression thresholds set to 0.56. For the genes in each quadrant, the top few GO biological process terms and Benjamini p-values are listed. For a complete list see S3 Table. Note that genes with high transcript levels in both human and mouse oocytes and placenta are enriched for many of the same functions as the genes with conserved high methylation across mammalian placentas (Fig 2A). (C) Venn diagram showing overlap of genes with high gene body methylation in mammalian placentas (see Fig 2A), high gene body methylation in human, mouse, and cow oocytes (see Fig 4B), and high expression in human and mouse placenta and oocytes (see A-B above). Mouse numbers are shown in parentheses. Note that the first two gene body methylation datasets only used Ensembl genes with orthologs across multiple mammalian species. The gene expression datasets used all RefSeq human and mouse genes. (TIFF) [file pgen.1005442.s017.tiff]
